# Costunolide normalizes neuroinflammation and improves neurogenesis deficits in a mouse model of depression through inhibiting microglial Akt/mTOR/NF-κB pathway

**DOI:** 10.1038/s41401-025-01506-w

**Published:** 2025-02-26

**Authors:** Shao-qi Zhang, Qiao Deng, Cheng Tian, Huan-huan Zhao, Li-ying Yang, Xin-wei Cheng, Guo-ping Wang, Dong Liu

**Affiliations:** 1 Institute of Pathology, Tongji Hospital, Tongji Medical College, Huazhong University of Science and Technology, Wuhan, 430030 China; 2 Department of Pathology, School of Basic Medicine, Tongji Medical College, Huazhong University of Science and Technology, Wuhan, 430030 China; 3 Department of Pharmacy, Tongji Hospital, Tongji Medical College, Huazhong University of Science and Technology, Wuhan, 430030 China; 4 Cancer Institute, Xuzhou Medical University, Xuzhou, 221004 China

**Keywords:** major depression, chronic stress, costunolide, microglia, mTOR, neurogenesis

## Abstract

Neuroinflammation is crucial for the pathogenesis of major depression. Preclinical studies have shown the potential of anti-inflammatory agents, specifically costunolide (COS), correlate with antidepressant effects. In this study, we investigated the molecular mechanisms underlying the antidepressant actions of COS. Chronic restraint stress (CRS) was induced in male mice. The mice were treated with either intra-DG injection of COS (5 μM, 1 μL per side) or COS (20 mg/kg, i.p.) for 1 week. We showed that administration of COS through the both routes significantly ameliorated the depressive-like behavior in CRS-exposed mice. Furthermore, administration of COS significantly improved chronic stress-induced adult hippocampal neurogenesis deficits in the mice through attenuating microglia-derived neuroinflammation. We demonstrated that COS (5 μM) exerted anti-neuroinflammatory effects in LPS-treated BV2 cells via inhibiting microglial Akt/mTOR/NF-κB pathway; inactivation of mTOR/NF-κB/IL-1β pathway was required for the pro-neurogenic action of COS in CRS-exposed mice. Our results reveal the antidepressant mechanism of COS that is normalizing neuroinflammation to improve neurogenesis deficits, supporting anti-inflammatory agents as a potential therapeutic strategy for depression.

## Introduction

Major depressive disorder (MDD) is a debilitating mental illness which inflicts over 350 million people worldwide [1, 2]. Although existing therapeutic antidepressants are widely used, slow onset, low rates of complete remission, and side effects limit the clinical applications, reflecting the intertwined pathogenesis of MDD. The novel diagnostic criteria for MDD emphasized that the neuroinflammation is a crucial cause for the pathophysiology of major depression [3]. Thus, identifying novel drug targets by exploring inflammatory mechanisms of depression becomes highly urgent.

Microglia, the tissue-resident macrophages of the brain, are essential regulators of neuroinflammation in response to chronic stress [4–6]. Lipopolysaccharide (LPS), a potent agent for excessive microglial activation, induces depressive-like behavior through neuroinflammation [7–10]. Moreover, neuroinflammation mediated by hyperactivated microglia in the brain, especially in the hippocampus, has been identified as a hallmark of MDD [4, 11]. Accordingly, anti-inflammatory treatments exhibited antidepressive effects in both animals and humans, emphasized as a potential strategy for improving the depressive symptoms of MDD patients. Chronic stress leads to microglial hyperactivation, secreting pro-inflammatory cytokines, which result in neuronal dysfunction and MDD [12]. Adult hippocampal neurogenesis (AHN), occurring in the subgranular zone of dentate gyrus (DG), exports newborn neurons throughout life of mammals [13–15]. Neuroimaging and post-mortem studies of untreated MDD patients have clarified a remarkable decline of AHN in DG [16, 17], while opportune neurogenesis is indispensable to the effectiveness of antidepressants [18, 19]. Recently, microglia hyperactivation has been revealed to confer AHN impairment to depressive-like behavior in mice [20–22]. Cell-cell interaction between microglia and neural stem cell in the DG under pathological conditions involves pro-inflammatory cytokines, such as interleukin 1β (IL-1β) [23, 24]. Meanwhile, IL-1β is an essential mediator of the anti-neurogenic and hopeless effects of stress in rodents and humans through its receptor [25, 26]. Inhibition of microglia-derived IL-1β improves AHN and depressive-like behavior [20, 27, 28]. Thus, exploring novel ways to rescue neuroinflammation-mediated disruption of AHN in the DG is essential for MDD treatment.

Costunolide (COS) is the major active ingredient of the traditional Chinese medicinal herb *Saussurea lappa*, which has potential therapeutic benefits in anti-inflammatory activity [29–32]. Recently, it has been identified that COS is capable of crossing the blood-brain barrier [33] and exerts antidepressant effects in mice [33] and zebrafish [34]. Whether the anti-neuroinflammatory activity of COS is involved in its antidepressant effects remains unclear. The mammalian target of rapamycin (mTOR) is a serine/threonine protein kinase that controls cellular homeostasis and immune responses, and dysregulation of mTOR signaling is strongly linked with neuroinflammation [35–37]. Nuclear factor kappa B (NF-κB), a classical transcriptional factor mediating the level of inflammatory genes, has been shown to play critical role in microglial activation and neuroinflammation, which can be inhibited by mTOR signaling [31]. Both mTOR and NF-κB signaling can be repressed by COS, implying that the mTOR/NF-κB signaling may be involved in the anti-inflammatory action of COS [38]. Moreover, Akt kinase plays a crucial role in mediating NF-κB-dependent inflammatory gene transcription [39, 40]. Upregulation of pro-inflammatory cytokines in microglia impaired hippocampal neurogenesis through Akt/mTOR/NF-κB signaling [41]. Considering that COS functions on inhibiting the production of IL-1β and IL-1β is a crucial mediator of AHN [42], whether COS ameliorates chronic stress-induced AHN deficits and depressive-like behavior through inhibiting the microglial Akt/mTOR/NF-κB signaling needs to be clarified.

In the present study, we found that COS ameliorated LPS- or chronic restraint stress-induced depressive-like behavior and microglia-mediated neuroinflammation in DG of male mice. COS alleviates neuroinflammation via inhibiting microglial Akt/mTOR/NF-κB pathway, further improving AHN deficits of DG. Furthermore, microglia-derived IL-1β signaling within DG functions as the key contributors for impairing AHN, which could be reversed by COS treatment. Our results support that COS ameliorates AHN impairment in DG by relieving microglia hyperactivation dependent on microglial Akt/mTOR/NF-κB pathway, eventually producing antidepressant effects.

## Materials and methods

### Animals

C57BL/6 J male mice (6–8 weeks old, Hubei BIONT Laboratory Animal Co, Ltd, Wuhan, China) were group-housed in Laboratory Animal Center of Tongji Medical College at room temperature (24 ± 2 °C) and under a 12 h-light/dark cycle with *ad libitum* access to food and water. All experiments on mice were allowed by the Animal Welfare Committee of Huazhong University of Science and Technology (project number 4175) and were used according to the guidelines in the ARRIVE guidelines 2.0 [43].

### Reagents

LPS (L2880) and BrdU (B5002) were purchased from Sigma (St Louis, MO, USA). COS (purity ≥ 98%, AB0612) was obtained from Alfa Biotechnology (Chengdu, China). IL-1β (#201-LB) was used from R&D System (MN, USA). MHY1485 (#HY-B0795, mTOR activators) was purchased from MedChemExpress (NJ, USA).

### Chronic restraint stress (CRS)

Chronic restraint stress, a conventional procedure of depression, was performed according to a previous study with minor modifications [44, 45]. Briefly, mice were restrained by using a 50-mL adjustable ventilate cylindrical restrainers. Mice were immobilized in the restrainers for 4 h once daily for 14 consecutive days.

### Sucrose preference test (SPT)

SPT was used to evaluate anhedonia of mice and performed according to previous research with slight alterations [46]. The experimental mice were habituated to two identical 50 mL bottles containing water or 1% sucrose solution during 24 h. After 12 h of water deprivation, mice were given access to two bottles containing water or 1% sucrose solution for 24 h. The position of two bottles was interchanged to avoid side preference, and the consumption of two bottles was weighed. Sucrose preference (%) was calculated by the percentage of sucrose consumption/total sucrose and water consumption.

### Tail suspension test (TST)

TST was used to evaluate despair of mice according to previous study [46]. Mice were habituated in the behavioral room for 1 h. The tip of the mouse tail was suspended on the top of a box 20 cm above the floor. ANY-maze software (Stoelting, WI, USA) was used to trace the immobility time during 6 min.

### Forced swim test (FST)

FST was performed according to the previous research with minor alterations [46]. After habituating in the behavioral room for 1 h, mice were put into a transparent cylinder (25 cm height) filled with clean water at room temperature (23 ± 2 °C) to swim during 6 min. ANY-maze software was used to trace the immobility time for the last 4 min.

### Splash test (ST)

ST was based on a published method with slight changes [47]. After habituating in the behavioral room for 1 h, mice were splashed on the back with 10% sucrose solution 3 times and then put into a housing cage individually for 5 min under red light (230 V, 15 W). The total time of grooming behavior was recorded.

### Open field test (OFT)

OFT was used to evaluate the locomotor abilities of mice and performed with minor modifications [46]. Mice were habituated in the behavioral room for 1 h and individually placed in an open field (45 cm× 45 cm× 45 cm). ANY-maze software was used to record the locomotor distance during 10 min.

### Cell culture

The BV2 cell line was performed followed by previous study [48] and routinely cultured by DMEM/F-12 (Gibco, 11320033), which was added with 10% fetal bovine serum (Gibco, 10091155), 1% penicillin-streptomycin (Gibco, 15140122) in a 5% CO_2_ incubator at 37 °C. Cultured BV2 cells were dissociated with trypsin (Gibco, 25200072) and plated on poly-*D*-lsysine-coated slides at a density of 5×10^4^ cells/well, and then incubated with LPS (100 ng/mL) or/and COS (5 μM) for 24 h. Finally, BV2 cells were harvested and performed in Western blotting or immunofluorescent analysis.

### Cellular viability analysis

Cellular viability was detected through a Cell Counting Kit-8 (CCK-8) assay (Apex Bio, TX, USA) followed by the operation instruction [46]. In brief, BV2 cells were plated in a 96-well plate at a density of 5000 cells/well and incubated with LPS (100 ng/mL), COS (5 μM), or MHY1485 (10 μM) for 24 h. CCK-8 was added, and the cellular viability was detected as the absorbance at 450 nm by using a microplate reader.

### Western blotting

Western blotting was performed as previous study [46]. Brain matrices (68707, RWD, Shenzhen, China) was used to obtain coronal brain slices (100 μm), which were dissected into brain region (DG) based on brain atlases of mice. Brain tissues or BV2 cells were homogenized in RIPA lysis buffer containing protease and phosphatase inhibitors (R0010, Solarbio, Beijing, China) and then mixed with 4× loading buffer at 95 °C for 10 min. 8% or 10% SDS-PAGE (P1200, Solarbio, Beijing, China) was used to separate protein extracts (30 μg) and then transferred to a nitrocellulose membrane (HATF00010, Millipore, MA, USA). The electrophoresis and transmembrane apparatus were obtained from BIO-RAD (1658033, CA, USA). Then, the membrane was incubated overnight at 4 °C with primary antibodies and corresponding secondary antibodies conjugated with HRP. The primary antibodies were applied as following: anti-mTOR (#ET1608-5, 1:1000 dilution), S6 antibodies (#HA600084, 1:1000 dilution), and anti-phosphor-S6 antibodies (#HA721275, 1:1000 dilution) were obtained from HUAbio (Hangzhou, China). Anti-Akt (#9272, 1:1000 dilution), anti-phospho-Akt (#9271, 1:1000 dilution), anti-NF-κB (#8242, 1:1000 dilution) and anti-phospho-NF-κB p65 antibodies (#3033, 1:1000 dilution) were purchased from Cell Signaling Technology (Boston, MA, USA). Anti-IL-1β antibody (#AF-401-NA, 1:500 dilution) was obtained from R&D (MN, USA). Anti-IL-6 (#GB11117, 1:1000 dilution) and anti-TNF-α (#GB115726, 1:1000 dilution) antibodies were purchased from Servicebio (Wuhan, China). Anti-β-actin (#81115-1-RR, 1:20,000 dilution) was used from Proteintech (Wuhan, China). The membranes were visualized by using ECL chemiluminescence reagent (1705060, BIO-RAD, CA, USA).

### Stereotaxic surgery

Mice were anaesthetized with urethane (1 g/kg, i.p.). For drug infusion, the stereotaxic apparatus (68803, RWD, Shenzhen, China) was used to bilaterally implant 22-gauge stainless steel guide cannulas (62203, RWD, Shenzhen, China) above the DG (AP =−2.00 mm; ML = ±1.50 mm; DV: ‒2.10 mm; relative to Bregma). After recovering from operation over 7 days, drugs, including COS (2.5, 5 μM, 1 μL per side), MHY1485 (10 μM, 1 μL per side), IL-1β (5 μg/kg, 1 μL per side) were injected into DG for 1 week.

### Immunofluorescent analysis

Mice were anaesthetized with urethane (1 g/kg, i.p.) and then perfused with 0.01 M phosphate buffer saline (PBS) followed by 4% paraformaldehyde. The brain was post-fixed in 4% paraformaldehyde overnight and dehydrated with gradient sucrose at 4 °C. A freezing microtome (CM1950, Leica, Wetzlar, Germany) was used to obtain coronal brain slices (30 μm) containing DG brain region. Similarly, BV2 cells were harvested and fixed in 4% paraformaldehyde for 30 min. For microglial staining, brain slices or BV2 cells were incubated in blocking solution containing 0.3% Triton X-100 and 5% bovine serum albumin in PBS for 1 h at room temperature. After washing in PBS, brain slices were incubated with primary antibodies overnight at 4 °C. The primary antibodies as following were used: Anti-CD68 (MCA1957, 1:500 dilution) was from Bio-Rad (CA, USA) and anti-Iba1 antibody (#019-19741, 1:500 dilution) was purchased from Wako (Tokyo, Japan). Anti-phosphor-S6 antibodies (#HA721275, 1:200 dilution) and anti-phospho-NF-κB p65 antibodies (#3033, 1:200 dilution) were seen above. Then, brain slices were incubated with secondary antibodies for 2 h at room temperature. For BrdU staining, brain slices were acidized with 1 M HCl for 10 min and 2 M HCl for 10 min at room temperature and then an additional 20 min at 37 °C. After washing in 0.1 M boric acid (pH 8.5), the following staining processes were same as previous described [46]. The primary antibodies were applied as following: anti-BrdU antibody (#ab6326, 1:1000 dilution) was purchased from Abcam (Cambridge, UK). Anti-DCX (#4604, 1:500 dilution) and anti-GFAP antibodies (#3670, 1:500 dilution) were purchased from Cell Signaling Technology (Boston, USA). Anti-Sox2 antibody (#AF2018, 1:100 dilution) was obtained from R&D Systems (MN, USA). Anti-NeuN antibodies (#26975-1-AP, 1:100 dilution) were obtained from Proteintech (Wuhan, China). Confocal images were visualized with a confocal laser scanning microscope (ZEISS, LSM900 Airyscan, Oberkochen, Germany).

### Analysis of images

For microglial analysis, number of CD68^+^ and Iba1^+^ cells was calculated by an ImageJ software (NIH, MD, USA). Three-dimensional (3D) images and morphometric analysis of DG microglia, such as soma size, length, and intersections were reconstructed and calculated by Imaris software 9.0 (Bitplane, Zurich, Switzerland) [49].

For analysis of cells type-specific markers, the quantification of BrdU^+^Sox2^+^GFAP^+^ radial glia-like cells (RGLs), BrdU^+^DCX^+^ neuroblasts, and BrdU^+^NeuN^+^ neurons in the granular layer of DG were calculated by an ImageJ software [46].

For analysis of dendritic branch, the DCX^+^ immature neurons of DG in a 100-μm-thick brain sections were visualized by a confocal microscope. The reconstructions of newborn neurons and length, intersections of dendrites were analyzed by ImageJ software (NIH, MD, USA) [50]. Dendritic segments 50–150 μm away from the soma were imaged from newborn neurons of DG.

### Statistical analysis

All data were presented as the mean ± SEM and performed by using GraphPad Prism 9.0 software (GraphPad, CA, USA). Statistical significance was determined by Student’s *t* test to analyze the statistical significance between two groups. Two-way ANOVA or repeated measures ANOVA followed by Sidak’s *post hoc* test between multiple groups and *P* < 0.05 was defined as statistical significance.

## Results

### COS ameliorates depressive-like behavior of male mice

**Fig. 1:**
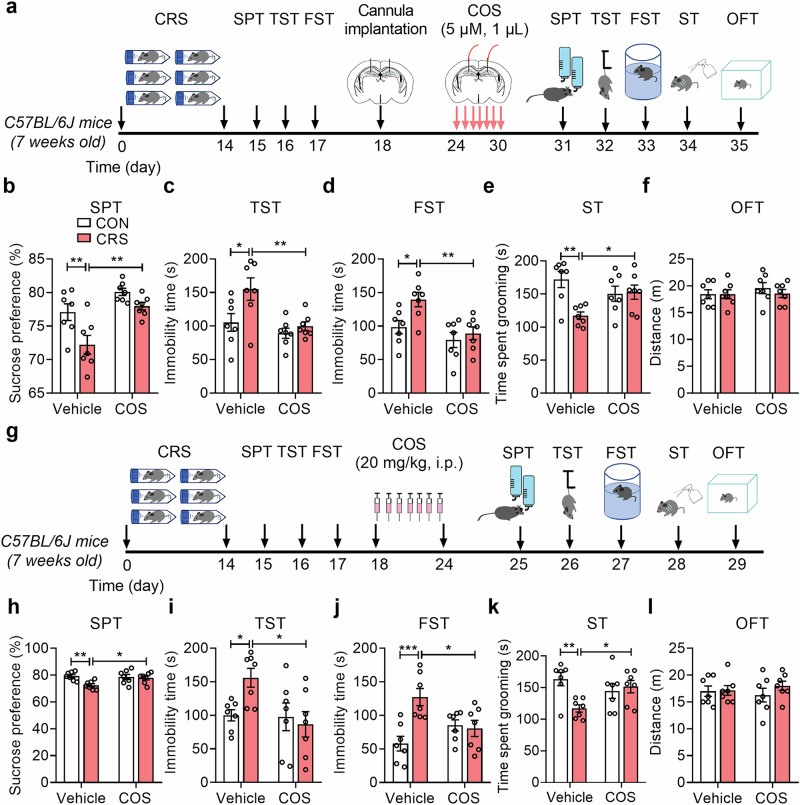
COS treatment ameliorates depressive-like behavior of male mice. **a** The experimental timeline. **b**–**e** Behavioral tests showed that intra-DG injection of COS (5 μM, 1 μL per side) ameliorated depressive-like behavior in CRS-exposed mice, including increased sucrose preference in SPT (**b**), decreased immobility time in TST (**c**) and FST (**d**), increased grooming time in ST (**e**) (*n* = 7 mice per group). **f** Open-field test showed that COS did not affect locomotor activity of mice (*n* = 7 mice per group). **g** The experimental timeline. **h–k** Behavioral tests showed that COS (20 mg/kg, i.p.) produced antidepressant action in CRS-exposed mice, including increased sucrose preference in SPT (**h**), decreased immobility time in TST (**i**) and FST (**j**), increased grooming time in ST (**k**) (*n* = 7 mice per group). **l** Open-field test showed that COS did not affect locomotor activity of mice (*n* = 7 mice per group). Data are presented as mean ± SEM, ^*^P < 0.05, ^**^P < 0.01, ^***^P < 0.001 by two-way ANOVA (**b**–**f**, **h**–**l**) followed by Sidak’s *post hoc* test. The statistical details can be found in Supplementary Table S1.

To clarify the antidepressant effect of COS in mice model of depression, we adopted CRS or LPS (0.1 mg/kg, i.p.) to induce depressive-like behavior of mice. After restraint stress of 4 h per day for 14 consecutive days of mice, forced swim test was performed after intra-DG injection with COS (2.5 μM or 5 μM, 1 μL per side) for 1 week (Supplementary Fig. S1a). We first observed that COS (5 μM, 1 μL per side) treatment for 1 week, but not COS (2.5 μM, 1 μL per side), decreased the immobility time in the FST of CRS-exposed mice compared with that of vehicle (Supplementary Fig. S1b, c), suggesting that COS (5 μM, 1 μL per side) treatment in DG produces antidepressant effect of mice. Furthermore, behavioral tests were performed after intra-DG injection (5 μM, 1 μL per side) of COS after CRS exposure (Fig. 1a). COS led to an increase in the sucrose preference of SPT (Fig. 1b), the decrease in the immobility time of TST (Fig. 1c) and FST (Fig. 1d), and significantly increased grooming time in the ST (Fig. 1e) of CRS-exposed mice compared with that of vehicle, while the locomotor activity was unaffected (Fig. 1f). Additionally, LPS exposure for 1 week induced depressive-like behavior [51], which was ameliorated by intra-DG injection of COS (5 μM, 1 μL per side) in DG for 1 week, including increased sucrose preference in the SPT, decreased immobility time in the TST and FST, and increased grooming time in the ST of LPS-exposed mice compared with that of vehicle (Supplementary Fig. S1d-h), indicating that COS produces antidepressant effects in CRS- and LPS-exposed mice.

Given that COS could penetrate blood-brain barrier [33], mice were administrated intraperitoneally with COS (5, 10 or 20 mg/kg, i.p.) for 1 week after CRS (Supplementary Fig. S1i). We found that COS (20 mg/kg, i.p.) significantly reduced immobility time in the FST of CRS-exposed mice compared with that of vehicle, while COS (5 mg/kg, i.p.) or COS (10 mg/kg, i.p.) did not decrease immobility time in the FST of CRS-exposed mice compared with that of vehicle (Supplementary Fig. S1j-l). Additionally, behavioral tests were applied after COS (20 mg/kg, i.p.) treatment for 1 week of CRS-exposed mice (Fig. 1g), and found that COS significantly increased sucrose preference in the SPT (Fig. 1h), reduced immobility time in the TST (Fig. 1i) and FST (Fig. 1j), and elevated grooming time in the ST (Fig. 1k) of CRS-exposed mice compared with that of vehicle. And the locomotor activity was not affected in all groups (Fig. 1l). Together, these results suggest that COS ameliorates depressive-like behavior of male mice.

### COS attenuates hyperactivation of microglia in DG induced by CRS

**Fig. 2:**
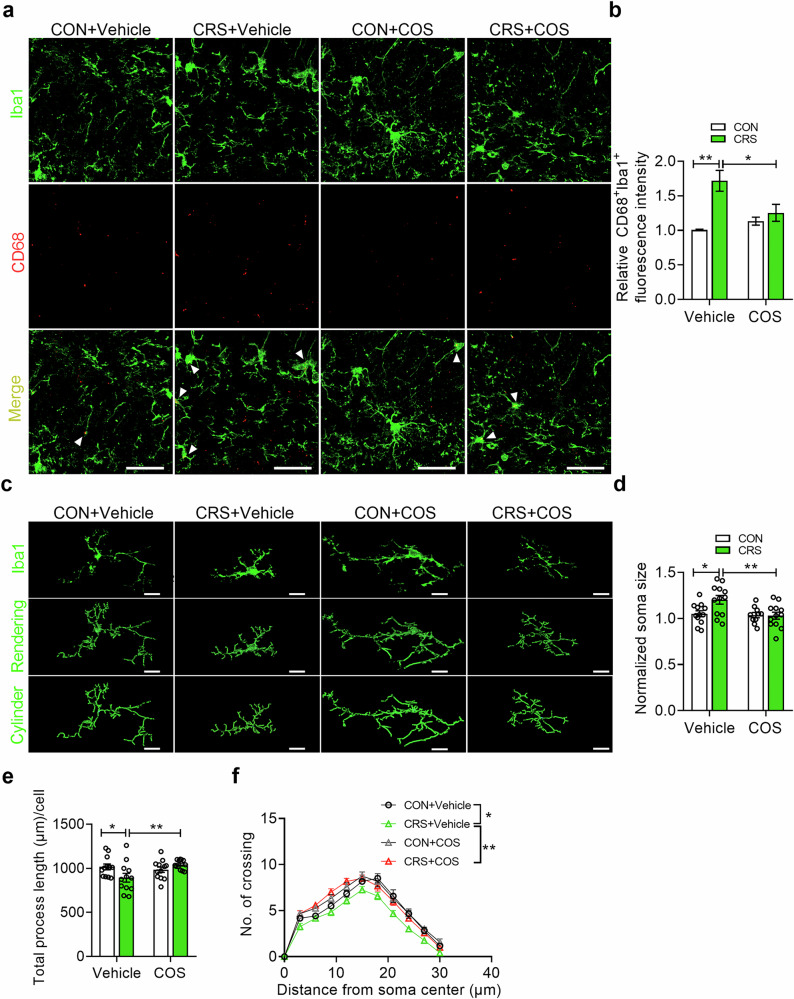
COS attenuates microglial hyperactivation in the DG induced by CRS. **a** Representative images of Iba1 (green) and CD68 (red) positive microglia in DG of control and CRS-exposed mice after vehicle or COS treatment. Scale bars, 30 μm. **b** Quantification of Iba1 and CD68 immunostaining in DG (*n* = 3 mice per group). **c** Representative images of Iba1 (top), 3D reconstruction (middle) and cylinder (bottom) of microglia in DG of control and CRS-exposed mice after vehicle or COS treatment. Scale bars, 10 μm. **d**–**f** Quantification of Iba1 positive cell soma size (**d**), total process length (**e**) and number of intersections (**f**) of microglia in DG (*n* = 10–12 cells from 3 mice per group). Data are presented as mean ± SEM, ^*^P < 0.05, ^**^P < 0.01 by two-way ANOVA (**b**, **d**, **e**), repeated measures ANOVA (**f**) followed by Sidak’s *post hoc* test. The statistical details can be found in Supplementary Table S1.

Emerging studies have demonstrated the role of microglial hyperactivation in response to chronic stress [52]. To explore the anti-neuroinflammatory action of COS, we adopted LPS (100 ng/mL) incubation in BV2 cells for 24 h to induce microglial activation. Immunofluorescent staining demonstrated that the intensity of Iba1 (a marker of microglia) or CD68 (a marker of phagocytic activity) in BV2 cells was increased by LPS incubation, which could be reversed by COS (5 μM) (Supplementary Fig. S2a, b), indicating that COS alleviated microglial activation induced by LPS in vitro. Then, we evaluated the effect on activated microglia of DG by CRS exposure by using CD68 and Iba1 (Fig. 2a). It was found that CRS increased the co-location intensity of CD68 and Iba1 in DG of CRS-exposed mice, which was reversed by COS treatment (5 μM, 1 μL per side) (Fig. 2b), suggesting that the activation of microglia induced by CRS was prevented by COS treatment.

We further analyzed the morphological changes of microglia in the DG of CRS-exposed mice after COS treatment (5 μM, 1 μL per side) (Fig. 2c), as it is well known to correlate closely with its activation state. Morphometric 3D measurements of microglia analysis revealed that the enlargement of soma volume and the decrease in length of microglial branches in DG of CRS-exposed mice compared with that of controls, which were reversed by COS treatment (Fig. 2d, e). Sholl analysis further indicated that a decreased microglial complexity of DG was induced by CRS, which was abolished by COS treatment compared with that of vehicle (Fig. 2f). These suggest that COS exerts anti-neuroinflammatory effect in CRS by inhibiting the hyperactivation of microglia in the DG.

### COS improves CRS-induced AHN deficits in DG

**Fig. 3:**
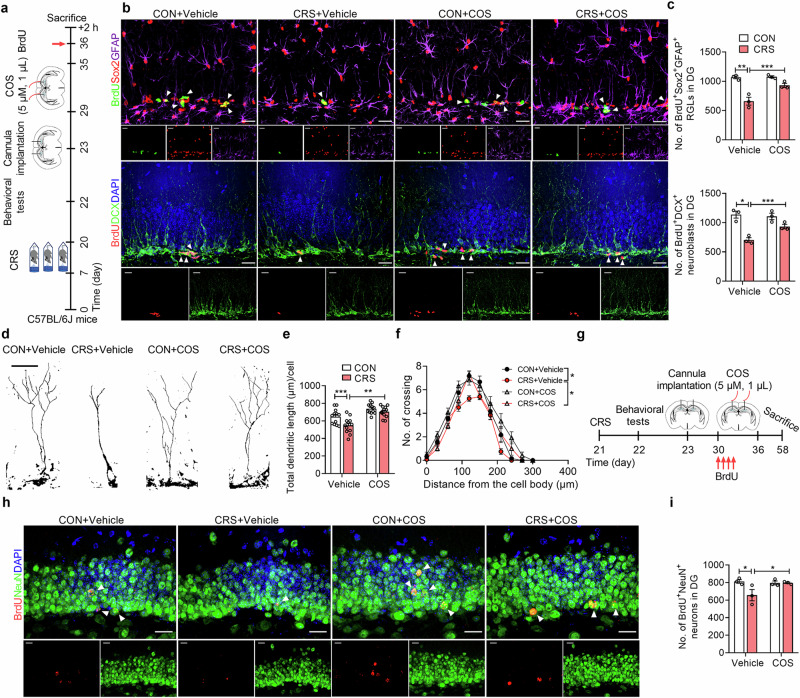
COS improves CRS-induced AHN deficits in DG. **a** The experimental timeline. **b** and **c** Representative images (**b**) and quantification (**c**) of BrdU^+^Sox2^+^GFAP^+^ RGLs and BrdU^+^DCX^+^ neuroblasts in DG of control and CRS-exposed mice after vehicle or COS treatment (*n* = 3 mice per group). White arrows indicate BrdU^+^ and marker^+^ cells. Scale bars, 20 μm. **d** Representative tracers of DCX^+^ immature neurons in DG of control and CRS-exposed mice after vehicle or COS treatment (*n* = 3 mice per group). Scale bars, 20 μm. **e**, and **f** Quantification of the dendritic length (**e**) and dendritic complexity (**f**) of DCX^+^ immature neurons in DG by Sholl analysis (*n* = 12 cells of 3 mice per group). **g** The experimental timeline. **h** and **i** Representative images (**h**) and quantification (**i**) of BrdU^+^NeuN^+^ newborn neurons in DG of control and CRS-exposed mice after vehicle or COS treatment (*n* = 12 cells of 3 mice per group). White arrows indicate the co-labeling cells. Scale bars, 20 μm. Data are presented as mean ± SEM, ^*^P < 0.05, ^**^P < 0.01, ^***^P < 0.001 by two-way ANOVA (**c**, **e**, **i**), repeated measures ANOVA (**f**) followed by Sidak’s *post hoc* test.

Given the detrimental impact of neuroinflammation in AHN impairment [53], we detected the role of COS on the proliferation of the neural stem cells with cell lineage-specific markers in CRS-exposed mice after injection with a 2-h BrdU (200 mg/kg, i.p.) pulse-chase (Fig. 3a). It was found that CRS resulted in a lower number of BrdU^+^Sox2^+^GFAP^+^ RGLs and BrdU^+^DCX^+^ neuroblasts of DG than that with controls (Supplementary Fig. S3a, b). And the reduced number of BrdU^+^Sox2^+^GFAP^+^ RGLs and BrdU^+^DCX^+^ neuroblasts of DG induced by CRS was prevented by COS treatment (5 μM, 1 μL per side) (Fig. 3b, c). We further applied DCX-labeling immature neurons and revealed that the decrease in total dendrite length and complexity of CRS-exposed mice was reversed by COS treatment (Fig. 3d–f), suggesting that COS ameliorates the defects in development of newborn neurons in DG induced by chronic stress.

To examine the role of COS on the generation of newborn neurons in CRS-exposed mice, mice were given BrdU (50 mg/kg, i.p.) injection twice a day for 2 days, and the immunofluorescences were measured 4 weeks later (Fig. 3g). It was revealed that CRS caused a lower number of BrdU^+^NeuN^+^ newborn neurons of DG than that with controls, while it was reversed by COS treatment (Fig. 3h, i).

These indicate that COS improves CRS-induced AHN deficits in DG.

### COS produces anti-neuroinflammatory effects via inhibiting microglial Akt/mTOR/NF-κB pathway

**Fig. 4:**
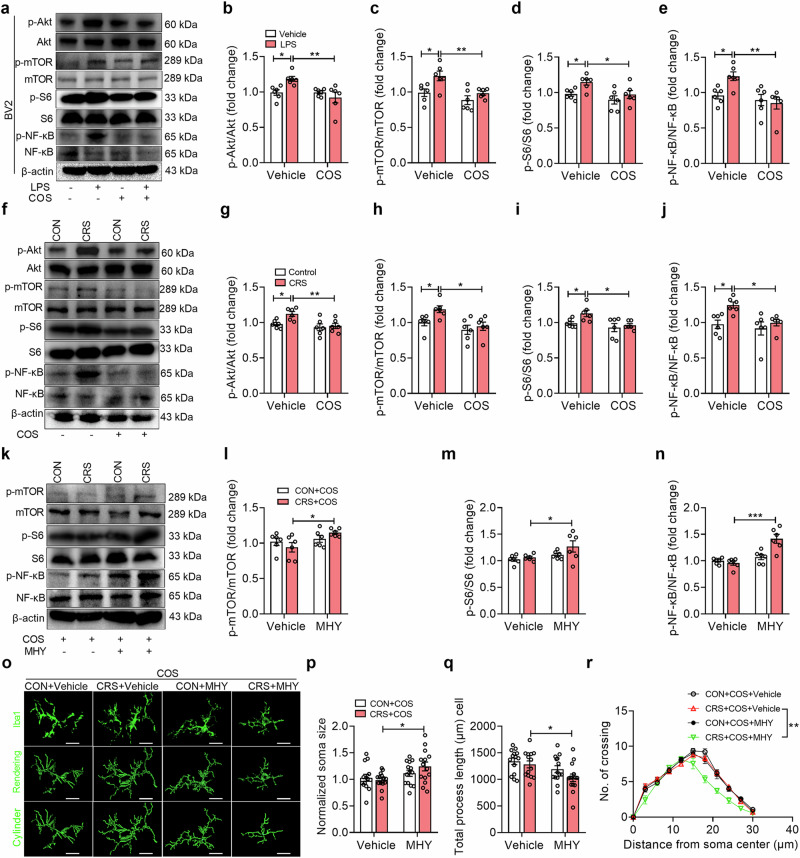
COS produces anti-neuroinflammatory effects via inhibiting microglial Akt/mTOR/NF-κB pathway. **a**–**e** Representative images (**a**) of Western blotting analysis and quantification showed that COS treatment prevented the increased phosphorylation protein expression of Akt (**b**), mTOR (**c**), S6 (**d**), NF-κB p65 (**e**) in BV2 cells induced by LPS exposure (*n* = 6 independent experiments per group). **f**–**j** Representative images (**f**) of Western blotting analysis and quantification showed that COS treatment prevented the increased phosphorylation protein expression of Akt (**g**), mTOR (**h**), S6 (**i**), NF-κB p65 (**j**) in DG induced by CRS (*n* = 6 mice per group). **k–n** Representative images (**k**) of Western blotting analysis and quantification showed that MHY1458 treatment (10 μM, 1 μL per side) blocked the decreased phosphorylation protein expression of mTOR (**l**), S6 (**m**), NF-κB p65 (**n**) in DG produced by COS treatment of CRS-exposed mice (*n* = 6 mice per group). **o** Representative images of Iba1 (top), three-dimensional (3D) reconstruction (middle) and cylinder (bottom) of microglia in DG (*n* = 12 cells from 3 mice per group). Scale bars, 10 μm. **p**–**r** Quantification showed that MHY1458 treatment blocked the decreased Iba1^+^ cell soma size (**p**) and the increased total process length (**q**) and number of intersections (**r**) of microglia in DG by COS treatment of CRS-exposed mice (*n* = 14 cells from 3 mice per group). Data are presented as mean ± SEM, ^*^P < 0.05, ^**^P < 0.01, ^***^P < 0.001 by two-way ANOVA (**b**–**e**, **g**–**j**, **l**–**n**, **p**, **q**), repeated measures ANOVA (**r**) followed by Sidak’s *post hoc* test.

Considering that COS suppresses the mTOR pathway in a dose-dependent manner [54] and the elevated inflammatory response confers to the activation of Akt/mTOR/NF-κB pathway, we observed that COS abolished the elevated immunofluorescent intensity of Iba1 and S6 phosphorylation (i.e., activation) of an mTOR substrate in LPS-treated BV2 cells (Supplementary Fig. S4a, b). To explore the underlying mechanisms of anti-neuroinflammatory effects induced by COS, we further found that the immunofluorescent intensity of Iba1 and NF-κB p65 phosphorylation in BV2 cells was increased after LPS incubation, which could be reversed by COS treatment (Supplementary Fig. S4c, d). Given that NF-κB generally enters the cell nucleus to play its role [55], immunofluorescent analysis showed that the immunofluorescent intensity of NF-κB p65 in nucleus was significantly increased by LPS incubations, which was reversed by COS treatment (Supplementary Fig. S4e, f). AKT is a direct target of COS [56, 57] and upstream signaling of mTOR [54, 58, 59]. Western blotting analysis showed that COS also reduced the increased protein expression of Akt phosphorylation, mTOR phosphorylation, and S6 phosphorylation in BV2 cells induced by LPS (Fig. 4a–d). And the increased protein expression of NF-κB p65 phosphorylation in BV2 cells after LPS incubation was reversed by COS (Fig. 4e). Similarly, we found that CRS increased the Akt phosphorylation, mTOR phosphorylation, S6 phosphorylation, and NF-κB p65 phosphorylation of DG compared with that of control, which was prevented by COS (Fig. 4f–j). These results indicate that COS produces anti-neuroinflammatory effects through inhibiting microglial Akt/mTOR/NF-κB signaling.

We further investigated whether the inactivation of mTOR/NF-κB signaling was required for COS’s anti-neuroinflammatory effect. Western blotting analysis showed that cell viability in BV2 cells was not affected by COS and mTOR activator MHY1485 incubations (10 μM) for 24 h (Supplementary Fig. S4g), and COS significantly decreased the S6 phosphorylation, and NF-κB p65 phosphorylation of BV2 cells incubated with LPS, which was reversed by MHY1485 treatment (Supplementary Fig. S4h–j). Meanwhile, it was found that intra-DG injection of MHY1485 (10 μM, 1 μL per side) abolished the decrease in mTOR phosphorylation, mTOR S6 phosphorylation and NF-κB p65 phosphorylation of CRS-exposed mice produced by COS (Fig. 4k–n). Additionally, MHY1485 reversed the decreased enlargement of soma volume, the elevated total process length, and the number of intersections of microglia in the DG, which were produced by COS treatment of CRS-exposed mice (Fig. 4o–r).

Together, inactivation of microglial Akt/mTOR/NF-κB pathway in DG is required for the anti-neuroinflammatory effects of COS.

### Inactivation of mTOR/NF-κB/IL-1β pathway is required for the pro-neurogenic action of COS

**Fig. 5:**
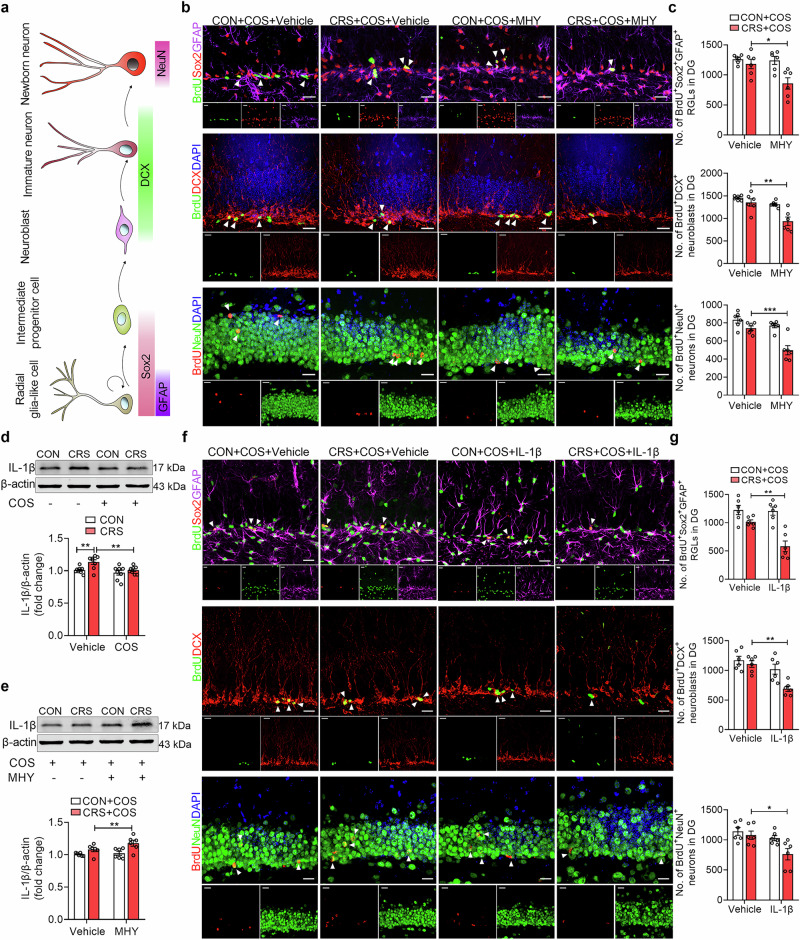
Inactivation of mTOR/NF-κB/IL-1β pathway is required for the pro-neurogenic action of COS. **a** Schematic paradigms of cell-lineage-specific markers in DG during AHN. **b**, **c** Representative images (**b**) and quantification (**c**) of BrdU^+^Sox2^+^GFAP^+^ RGLs, BrdU^+^DCX^+^ neuroblasts, and BrdU^+^NeuN^+^ newborn neurons in DG (*n* = 6 mice per group). White arrows indicate BrdU^+^ and Marker^+^ cells. Scale bars, 20 μm. **d** Treatment with COS (5 μM, 1 μL per side) reversed the increased IL-1β protein expression in DG of CRS-exposed mice (*n* = 8 mice per group). **e** Intra-DG injection of MHY1485 (10 μM, 1 μL per side) abolished the decreased IL-1β protein expression of CRS-exposed mice produced by COS (*n* = 6 mice per group). **f**, **g** Representative images (**f**) and quantification (**g**) of BrdU^+^Sox2^+^GFAP^+^ RGLs, BrdU^+^DCX^+^ neuroblasts, and BrdU^+^NeuN^+^ newborn neurons in DG (*n* = 6 mice per group). White arrows indicate BrdU^+^ and Marker^+^ cells. Scale bars, 20 μm. Data are presented as mean ± SEM, ^*^P < 0.05, ^**^P < 0.01, ^***^P < 0.001 by two-way ANOVA (**c**, **d**, **e**, **g**) followed by Sidak’s *post hoc* test.

Considering the pivotal role of the mTOR/NF-κB pathway in mediating the anti-neuroinflammatory effect of COS, we sought to investigate whether the mechanism could underlie its potential for ameliorating deficits in AHN. By using MHY1485 (10 μM, 1 μL per side) to evaluate the pro-neurogenic effect of COS, and found that MHY1485 abolished the increased number of BrdU^+^Sox2^+^GFAP^+^ RGLs, BrdU^+^DCX^+^ neuroblasts, and BrdU^+^NeuN^+^ neurons in DG of CRS-exposed mice after COS treatment (Fig. 5a-c), suggesting that mTOR activator abolishes the pro-neurogenic effect of COS treatment.

Pro-inflammatory protein IL-1β impairs human hippocampal neurogenesis [24], and COS inhibits the production of IL-1β [60, 61]. Accordingly, we wondered whether IL-1β as a key downstream of microglial activation in DG to modulate AHN. Then, we measured the protein level of IL-1β in DG. It was found that CRS significantly increased the protein expression of IL-1β in DG, which was prevented by COS treatment (Fig. 5d). While it was found that COS treatment at dosage of 5 μM did not affect the elevated protein level of IL-6 and tumor necrosis factor-α (TNF-α) induced by CRS (Supplementary Fig. S5a–c), which is similar with previous study [60]. MHY1485 abolished the decreased protein level of IL-1β in DG of CRS-exposed mice produced by COS treatment compared with that of vehicle (Fig. 5e). These findings suggest that mTOR/NF-κB pathway may serve as an upstream regulator of IL-1β in DG, while COS inhibits the activation of this pathway induced by CRS. Furthermore, administration of IL-1β (5 μg/kg, 1 μL per side) into DG abolished the increased number of BrdU^+^Sox2^+^GFAP^+^ RGLs, BrdU^+^DCX^+^ neuroblasts, and BrdU^+^NeuN^+^ newborn neurons in DG by COS treatment of CRS-exposed mice (Fig. 5f, g), indicating that IL-1β abrogates the protective effect on AHN deficits in DG under chronic stress after COS treatment.

Thus, these results suggest that COS possesses the ability to suppress the activation of microglial mTOR/NF-κB/IL-1β pathway in order to alleviate neuroinflammation and restore AHN deficits induced by CRS.

### Inhibition mTOR/NF-κB/IL-1β pathway confers to the antidepressant effects of COS

**Fig. 6:**
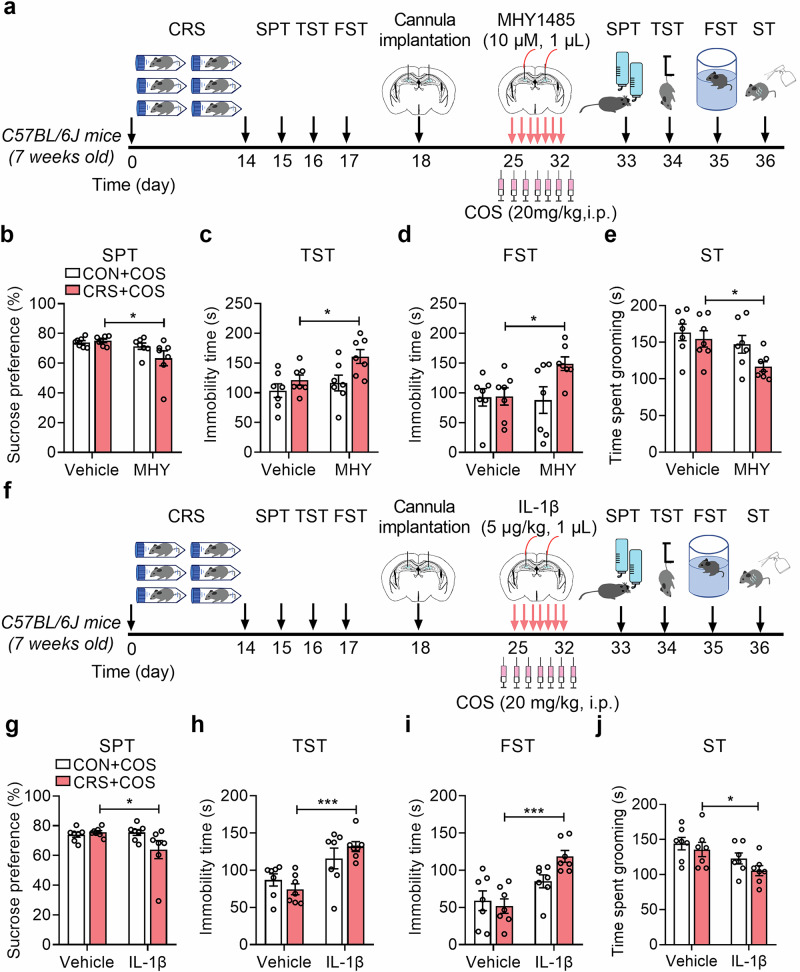
Inhibition mTOR/NF-κB/IL-1β pathway confers to the antidepressant effects of COS. **a** The experimental timeline. **b**–**e** Behavioral tests showed that MHY1485 (10 μM, 1 μL per side) reversed the antidepressant effects produced by COS (20 mg/kg, i.p.) in CRS-exposed mice, including decreased sucrose preference in SPT (**b**), increased immobile time in TST (**c**) and FST (**d**), and decreased grooming time in ST (**e**) (*n* = 7 mice per group). **f** The experimental timeline. **g-j** Behavioral test showed that IL-1β (5 μg/kg, 1 μL per side) abolished antidepressant effects induced by COS in CRS-exposed mice, including decreased sucrose preference in SPT (**g**), increased immobile time in TST (**h**) and FST (**i**), and decreased grooming time in ST (**j**) (*n* = 7 mice per group). Data are presented as mean ± SEM, ^*^P < 0.05, ^***^P < 0.001 by two-way ANOVA (**b**–**e**, **g**–**j**) followed by Sidak’s *post hoc* test.

To further investigate the involvement of mTOR/NF-κB/IL-1β pathway in the antidepressant effects of COS, the pharmacological methods were applied that COS treatment (20 mg/kg, i.p.) and infusion of vehicle or MHY1485 (10 μM, 1 μL per side) into DG of control or CRS-exposed mice (Fig. 6a). It was found that MHY1485 abolished the antidepressant effects of CRS-exposed mice produced by COS treatment, including the reduced sucrose preference in SPT, the elevated immobility time in TST and FST, and the decreased grooming time in ST of CRS + COS + MHY group (Fig. 6b–e). Similarly, MHY1485 abrogated the antidepressant effects of CRS-exposed mice produced by intra-injection of COS (5 μM, 1 μL per side) into DG (Supplementary Fig. S6a–e). These suggest that activation of mTOR in DG can weaken the antidepressant effects of COS treatment.

Then, intra-DG injection of IL-1β (5 μg/kg, 1 μL per side) was used to detect the antidepressant effects of CRS-exposed mice after COS treatment (20 mg/kg, i.p.) (Fig. 6f). We observed that IL-1β abrogated the increase in sucrose preference of SPT, the decrease in immobility time of TST and FST, and the increase in grooming time of ST of CRS-exposed mice produced by COS treatment (Fig. 6g–j). Meanwhile, IL-1β prevented the antidepressant effects of CRS-exposed mice produced by intra-injection of COS (5 μM, 1 μL per side) into DG (Supplementary Fig. S6f–j). This indicates that IL-1β functions as a pivotal mediator in the response to COS-mediated antidepressant effects.

Collectively, these findings demonstrate that the microglial Akt/mTOR/NF-κB/IL-1β pathway is responsible for the antidepressant effects of COS in an AHN-dependent manner.

## Discussion

**Fig. 7:**
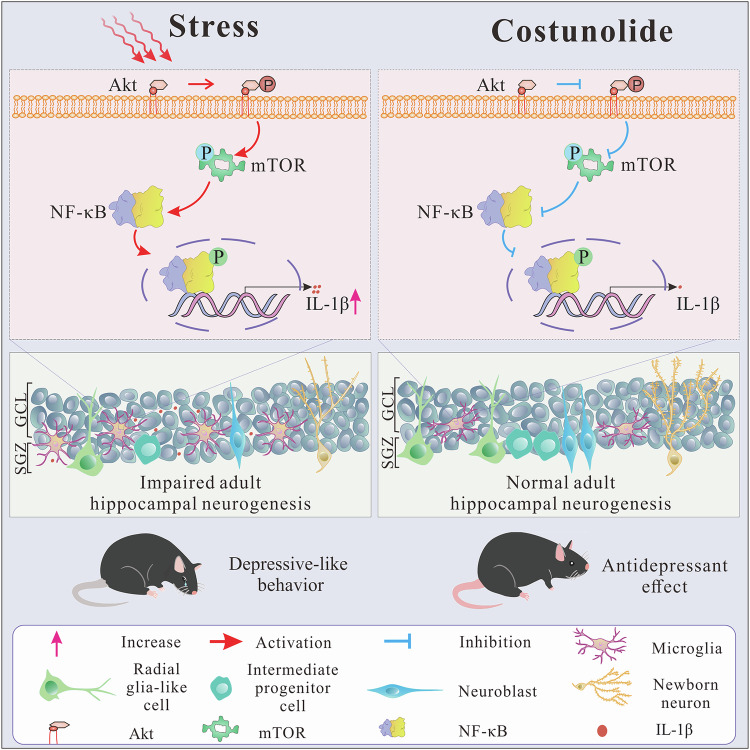
Schematic representation of the microglia activation-specific mechanism of COS in mediating the impairment of AHN in chronic stress-induced depression. COS ameliorates IL-1β-mediated neurogenesis deficits of DG via inhibiting microglial Akt/mTOR/NF-κB-dependent neuroinflammation, further improving chronic stress-induced depressive-like behavior.

In the present study, we elucidated a previously unrecognized molecular mechanism underlying the antidepressant effect of COS through neuroinflammation-mediated neurogenesis. Our findings demonstrated that COS exerted antidepressant effects by inhibiting microglial Akt/mTOR/NF-κB pathway, followed by ameliorating microglial hyperactivation coupled with AHN defects in the DG (Fig. 7). These results suggested that COS may serve as a potential therapeutic agent against chronic stress-induced depressive-like behavior.

COS plays a pivotal therapeutic role in for chronic inflammatory disease, such as atherosclerotic diseases [60]. Emerging evidence have reported that chronic stress induces depressive-like behavior through neuroinflammation [62–64]. The process of neuroinflammation results mainly from microglial activation [65, 66]. In the present study, it was found that COS alleviated microglial activation and morphological dysfunction in DG induced by LPS or CRS. These indicate the potentially therapeutic candidate of anti-neuroinflammation for depression. Microglial hyperactivation leads to pro-inflammatory cytokines secretion, further contributing to neuroinflammation-related pathological states [67, 68]. Although COS can reduce IL-6 and TNF-α, there were dose difference of COS, such as higher doses of COS or LPS [29, 69, 70]. Previous studies have reported that COS (5 μM) treatment reduces the protein expression of IL-1β, but not IL-6 and TNF-α, in a dose-dependent manner [60]. Our results demonstrated that COS reversed the increased expression of IL-1β in DG induced by CRS, thereby ameliorating CRS-induced neuroinflammation and depressive-like behavior. These suggest that pro-inflammatory factor IL-1β serves as a crucial mediator for the antidepressant effects of COS. Previous study has reported that COS produces antidepressant action in mice by increasing 5-hydroxytryptamine levels in the hippocampus [33], suggesting hippocampus as an antidepressant target region of COS. Meanwhile, it was found that intra-DG infusion of IL-1β abolished the antidepressant effect produced by COS. Collectively, our results imply that anti-neuroinflammation in the DG is critical for the antidepressant action of COS.

Previous reports have identified the targeted interaction between COS and NF-κB subunit proteins [71]. Specifically, COS alleviates inflammation in atherosclerosis by inhibiting NF-κB, a classical transcriptional factor that regulates the expression of many inflammatory genes [31]. Numerous evidence has demonstrated that the viral role of Akt/mTOR and mTOR/NF-κB signaling in chronic stress-induced neuroinflammation, respectively [36, 72], but fewer study identified whether the Akt/mTOR/NF-κB signaling pathway is involved in this process. Recently, Ravizza et al. has reported the mTOR signaling have been recognized as critical pathways in neuroinflammation [37], implying that mTOR may be the crucial hub mediating Akt/mTOR/NF-κB signaling-induced neuroinflammation under chronic stress condition. A recent study has shown that Akt/mTOR/NF-κB signaling pathway plays a detrimental role in the neurogenesis [41] and COS also suppresses phosphorylation of mTOR [54]. These indicate that COS may produce anti-inflammatory effects by inhibiting microglia and rescuing microglial morphology in DG through Akt/mTOR/NF-κB signaling pathway. In our study, we observed that incubation with COS prevented the increased protein expression of Akt phosphorylation, mTOR phosphorylation, S6 phosphorylation, and NF-κB p65 phosphorylation in BV2 cells induced by LPS incubation. Moreover, we found that mTOR activator MHY1485 abolished the activation of NF-κB signaling in DG. These indicate that COS inhibits the microglial Akt/mTOR/NF-κB pathway to alleviate neuroinflammation. Additionally, the activation of Akt/mTOR/NF-κB signaling in DG abolished the antidepressant effect of COS on CRS-exposed mice. Although NF-κB-dependent microglial activation has been widely reported, our result provides a novel molecular mechanism of microglial Akt/mTOR/NF-κB signaling pathway in DG for the anti-inflammatory effect of COS.

Although extensive evidence indicates that microglia-mediated neuroinflammation as a potential contributor to adult neurogenesis impairment in DG [21, 73, 74], the molecular mechanism remains to be uncovered. IL-1β has a potently anti-neurogenic effect in the human hippocampus, which is consistent with that in rodent models where IL-1 receptors are highly expressed in the neurogenic niche of DG [25]. In the present study, we found that COS reversed the increased level of IL-1β in DG induced by CRS. Additionally, it was found that COS ameliorated AHN deficits induced by CRS. Intra-DG injection of IL-1β abolished the therapeutic effect of COS on AHN deficits induced by CRS, suggesting that microglia-derived IL-1β in the DG functioned as the key mediators for promoting AHN by COS. Similarly, infusion of IL-1β into DG reversed the antidepressant effect of COS on CRS-exposed mice, suggesting that IL-1β signaling underlies the antidepressant effect of COS in AHN-dependent manner. More importantly, we found that mTOR activator MHY1485 could abolish the inhibitory effect of COS on IL-1β protein level in DG of CRS-exposed mice. Hence, IL-1β signaling may be a possible bridge mechanism between Akt/mTOR/NF-κB-mediated microglial hyperactivation and neurogenesis impairment in DG seen in mice model of depression.

## Conclusion

In summary, to the best of our knowledge, we present here evidence suggesting that COS alleviates neuroinflammation and AHN deficits in chronic stress-induced depressive-like behavior via inhibiting the microglial Akt/mTOR/NF-κB/IL-1β signaling pathway. Thus, our findings provide insight into the key therapeutic effect of COS and novel molecular mechanism of microglial Akt/mTOR/NF-κB/IL-1β signaling pathway for the treatment of major depression.

## Supplementary information

Supplementary information
