# Supplementary material for: Costunolide normalizes neuroinflammation and improves neurogenesis deficits in a mouse model of depression through inhibiting microglial Akt/mTOR/NF-κB pathway
Source: Acta Pharmacol Sin. 2025 Feb 26;46(7):1864–76. doi: 10.1038/s41401-025-01506-w (PMC12205059; doi:10.1038/s41401-025-01506-w)
Supplement: Supplementary file 1 — Supplementary information [file 41401_2025_1506_MOESM1_ESM.docx]

**Supplementary figures**


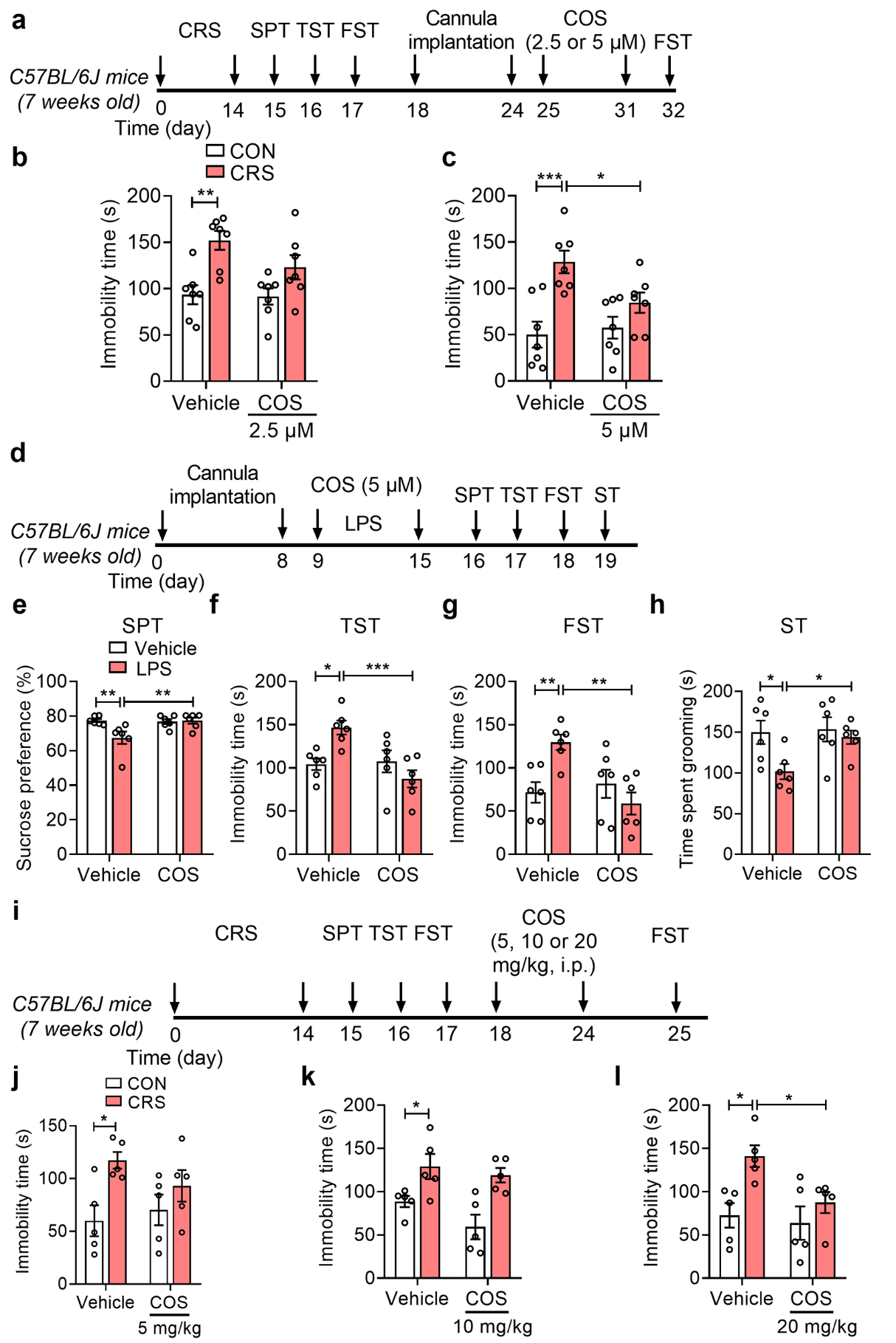


**Supplementary information, Fig. S1 COS ameliorates depressive-like behavior of male mice. a** Experimental paradigms for CRS and behavioral tests. **b** Behavioral tests of FST in control and CRS-exposed mice with COS treatment (2.5 μM, 1 μL per side) (*n* = 7 mice per group). **c** Behavioral tests of FST in control and CRS-exposed mice with COS treatment (5 μM, 1 μL per side) (*n* = 7 mice per group). **d** Experimental paradigms for LPS exposure and behavioral tests. **e-h** Depressive-like behavior in CRS-exposed mice, as measured by the sucrose preference in SPT (**e**), and the immobility time in TST (**f**) and FST (**g**), grooming time in ST (**h**) (*n* = 7 mice per group). **i** Experimental paradigms for COS treatment (i.p.). **j-l** Behavioral tests showed that COS (5 mg/kg, i.p.) (**j**) or COS (10 mg/kg, i.p.) (**k**) did not affect the increased immobility time in FST induced by CRS, but COS (20 mg/kg, i.p.) (**l**) decreased immobility time in FST in CRS-exposed mice compared with vehicle (*n* = 5 mice per group). Data are presented as mean ± SEM, ^*^*P* < 0.05, ^**^*P* < 0.01, ^***^*P* < 0.001 by two-way ANOVA (**b**, **c**, **e-h, j-l**) followed by Sidak’s *post hoc* test. The statistical details can be found in Supplementary Table S1.


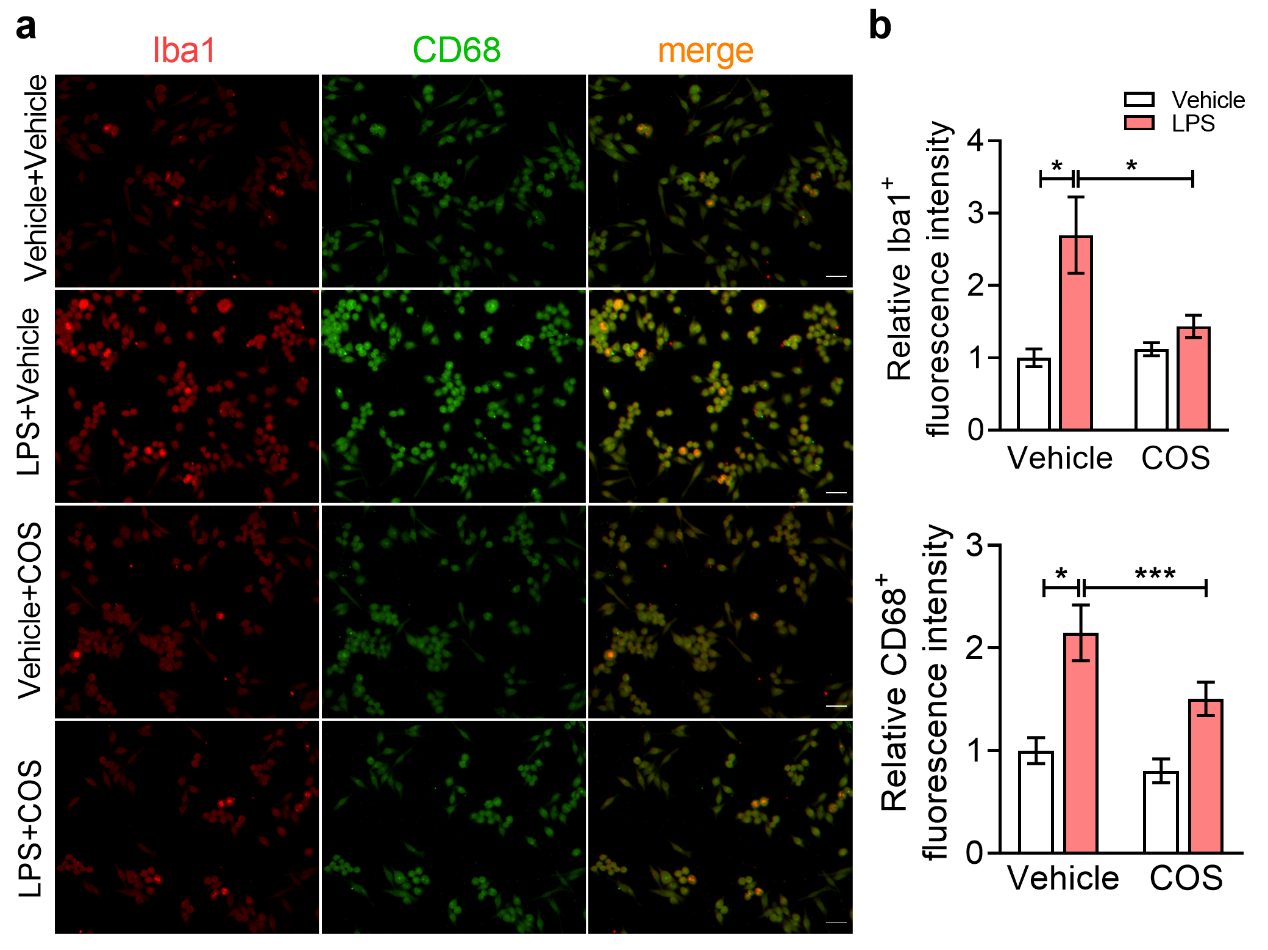


**Supplementary information, Fig. S2 COS alleviates microglial activation induced by LPS in BV2 cells. a** Representative image of Iba1 (red) and CD68 (green) positive microglia in BV2 cells with vehicle and LPS incubation (100 ng/mL) after vehicle or COS treatment. Scale bars, 20 μm. **b** Quantification analysis showed that COS prevented the increased Iba1 and CD68 immunostaining in BV2 cells from LPS incubation (*n* = 15 independent experiments per group). Data are presented as mean ± SEM, ^*^*P* < 0.05, ^***^*P* < 0.001 by two-way ANOVA test (**b**) followed by Sidak’s *post hoc* test. The statistical details can be found in Supplementary Table S1.


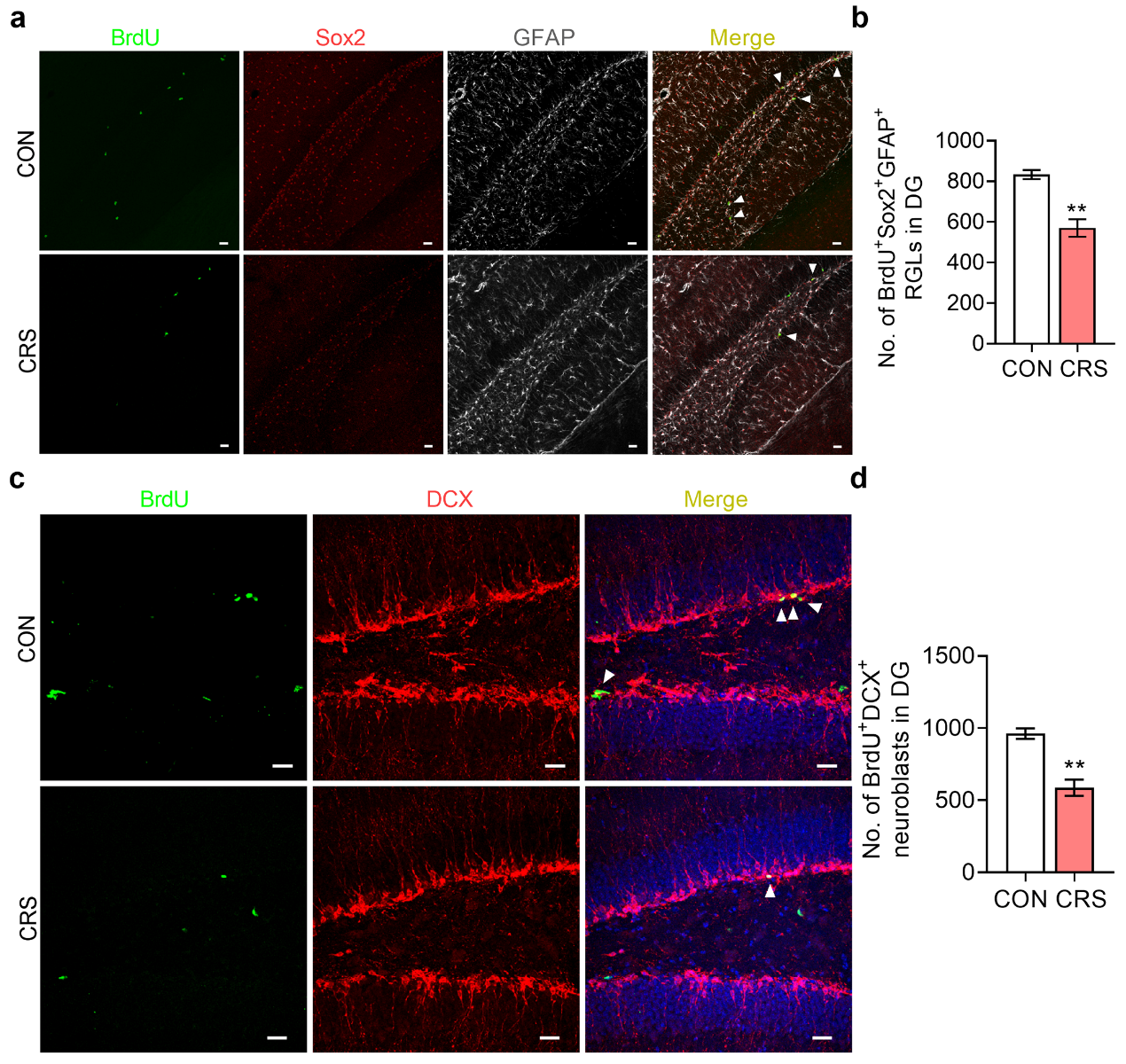


**Supplementary information, Fig. S3 CRS impairs AHN of DG. a-b** Representative images (**a**) and quantification (**b**) of BrdU^+^Sox2^+^GFAP^+^ RGLs in DG of control and CRS-exposed mice after vehicle or COS treatment (*n* = 3 mice per group). White arrows indicate BrdU^+^ and marker^+^ cells. Scale bars, 20 μm. **c and d** Representative images (**c**) and quantification (**d**) of BrdU^+^DCX^+^ neuroblasts in DG of control and CRS-exposed mice after vehicle or COS treatment (*n* = 3 mice per group). White arrows indicate BrdU^+^ and marker^+^ cells. Scale bars, 20 μm. Data are presented as mean ± SEM, ^**^*P* < 0.01 by Unpaired *t* test (**b, d**). The statistical details can be found in Supplementary Table S1.


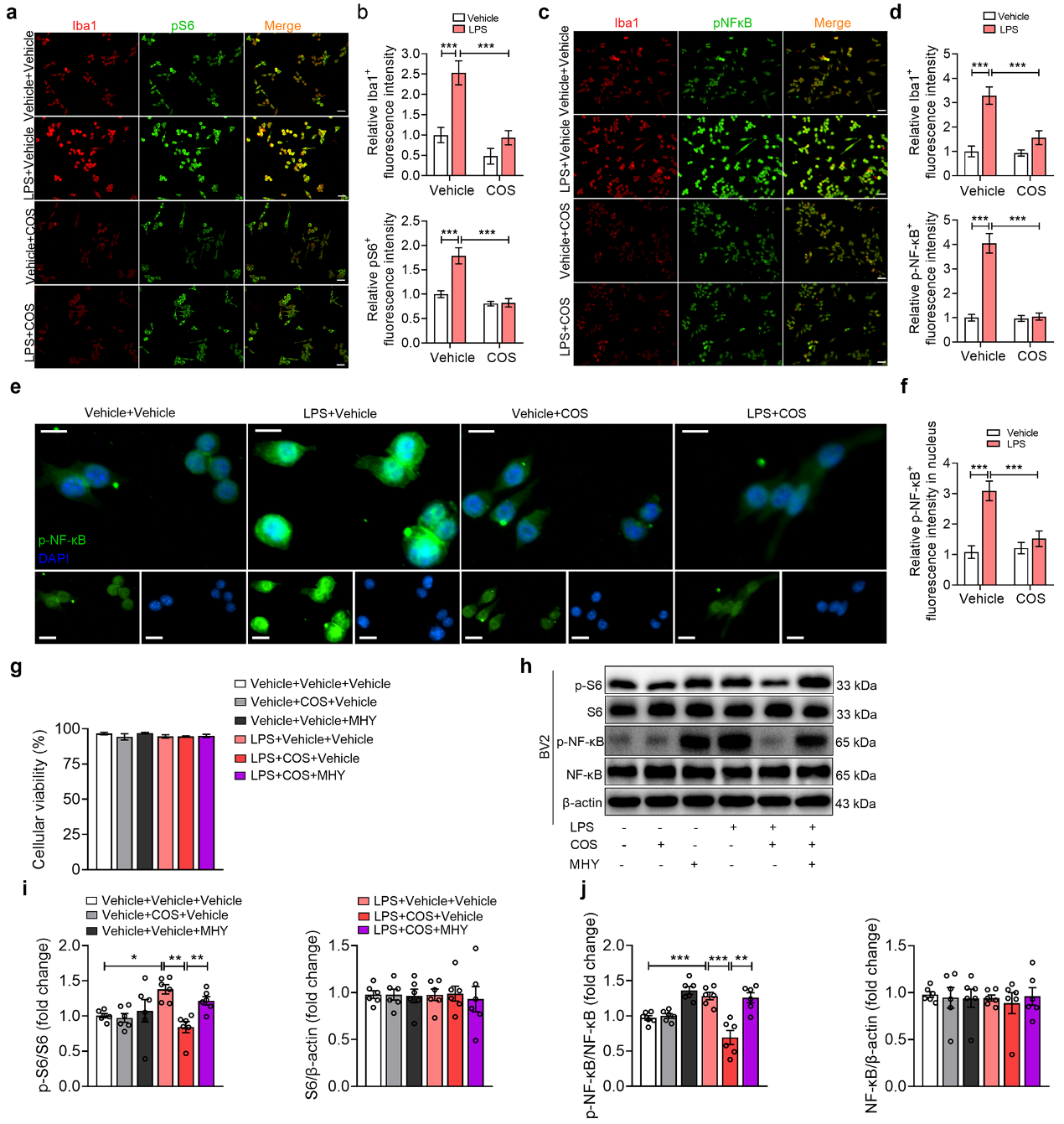


**Supplementary information, Fig. S4 COS inhibits mTOR/NF-κB phosphorylation in BV2 cells. a** Representative image of Iba1 immunostaining (red) and p-S6 (green) in BV2 cells of vehicle and LPS incubation after vehicle or COS treatment. Scale bars, 20 μm. **b** Quantification showed that COS treatment prevented the increased immunofluorescence intensity for Iba1 and p-S6 in BV2 cells induced by LPS exposure (*n* = 15 independent experiments per group). **c** Representative images of Iba1 immunostaining (red) and NF-κB p65 (green) in BV2 cells of vehicle and LPS incubation after vehicle or COS treatment. Scale bars, 20 μm. **d** Quantification showed that COS treatment prevented the increased immunofluorescence intensity for Iba1 and NF-κB p65 in BV2 cells induced by LPS exposure (*n* = 15 independent experiments per group). **e** Representative images of NF-κB p65 (green) and DAPI (blue) in BV2 cells of vehicle and LPS incubation after vehicle or COS treatment. Scale bars, 20 μm. **f** Quantification showed that COS treatment prevented the increased immunofluorescence intensity for NF-κB p65 and DAPI in BV2 cells induced by LPS exposure (*n* = 15 independent experiments per group). **g** Quantification showed that the cellular viability of BV2 cells was not affected by COS and MHY1485 treatment (*n* = 6 independent experiments per group). **h-j** Representative images (**h**) of Western blotting analysis and quantification showed that MHY1485 treatment reversed the decreased phosphorylation protein expression of S6 (**i**), NF-κB p65 (**j**) in BV2 cells induced by COS treatment under LPS exposure (*n* = 6 independent experiments per group). Data are presented as mean ± SEM, ^*^*P* < 0.05, ^**^*P* < 0.01, ^***^*P* < 0.001 by two-way ANOVA **(b**, **d, f, g, i, j)** followed by Sidak’s *post hoc* test. The statistical details can be found in Supplementary Table S1.


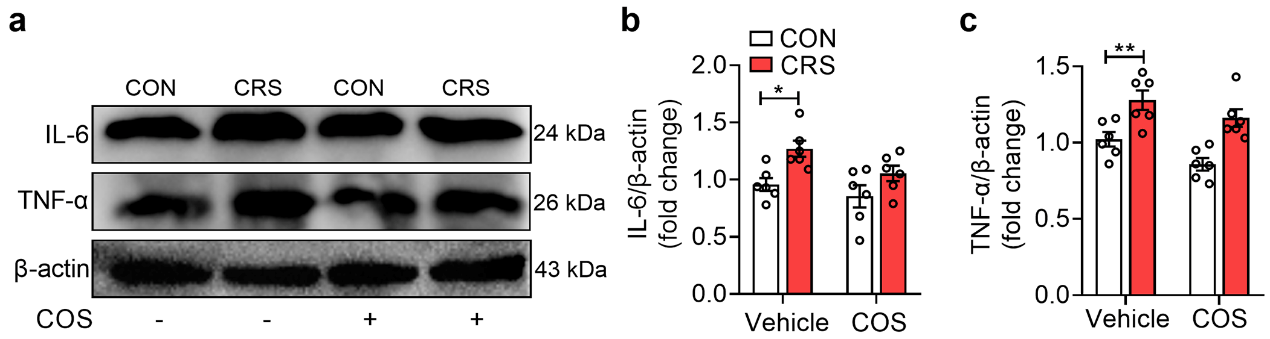


**Supplementary information, Fig. S5 COS rescues the increased expression of IL-6 and TNF-α in DG under chronic stress.** **a-c** Representative images (**a**) of Western blotting and quantification showed that COS treatment did not affect the increased protein expression of IL-6 (**b**) and TNF-α (**c**) in DG of CRS-exposed mice (*n* = 6 mice per group). Data are presented as mean ± SEM, ^*^*P* < 0.05, ^**^*P* < 0.01 by two-way ANOVA **(b**, **c)** followed by Sidak’s *post hoc* test. The statistical details can be found in Supplementary Table S1.


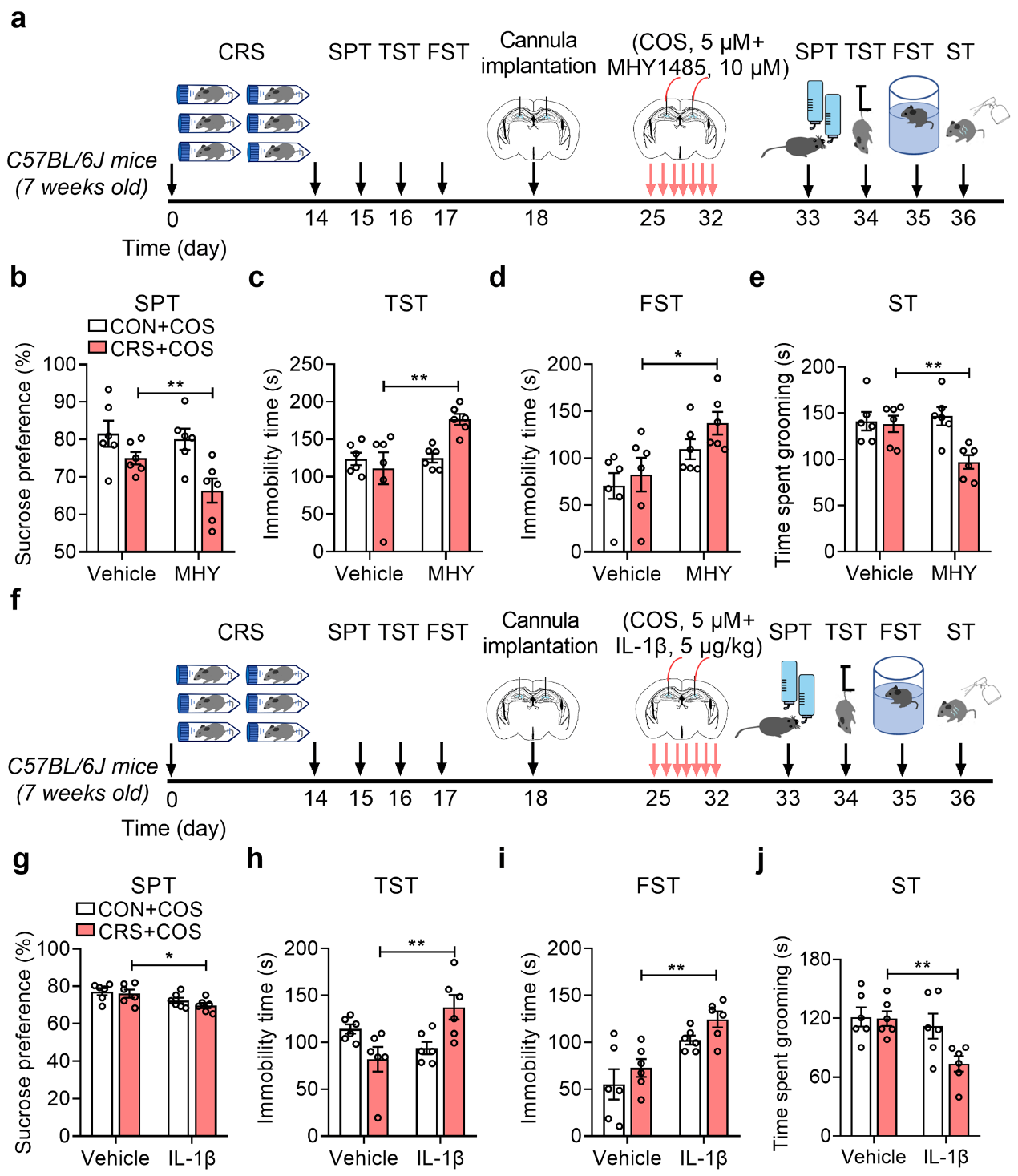


**Supplementary information, Fig S6 Intra-DG injection of MHY1485 or IL-1β abolishes the antidepressant effects of COS treatment in DG.** **a** The experimental timeline. **b-e** Behavioral tests showed that MHY1485 (10 μM, 1 μL per side) reversed the antidepressant effects produced by COS (5 μM, 1 μL per side) treatment in DG of CRS-exposed mice, including decreased sucrose preference in SPT (**b**), increased immobile time in TST (**c)** and FST (**d**), and decreased grooming time in ST (**e**) (*n* = 6 mice per group). **f** The experimental timeline. **g-j** Behavioral test showed that IL-1β (5 μg/kg, 1 μL per side) abolished antidepressant effects induced by COS (5 μM, 1 μL per side) treatment in DG of CRS-exposed mice, including decreased sucrose preference in SPT (**g**), increased immobile time in TST (**h**) and FST (**i**), and decreased grooming time in ST (**j**) (*n* = 6 mice per group). Data are presented as mean ± SEM, ^*^*P* < 0.05, ^**^*P* < 0.01 by two-way ANOVA **(b-e, g-j**) followed by Sidak’s *post hoc* test. The statistical details can be found in Supplementary Table S1.


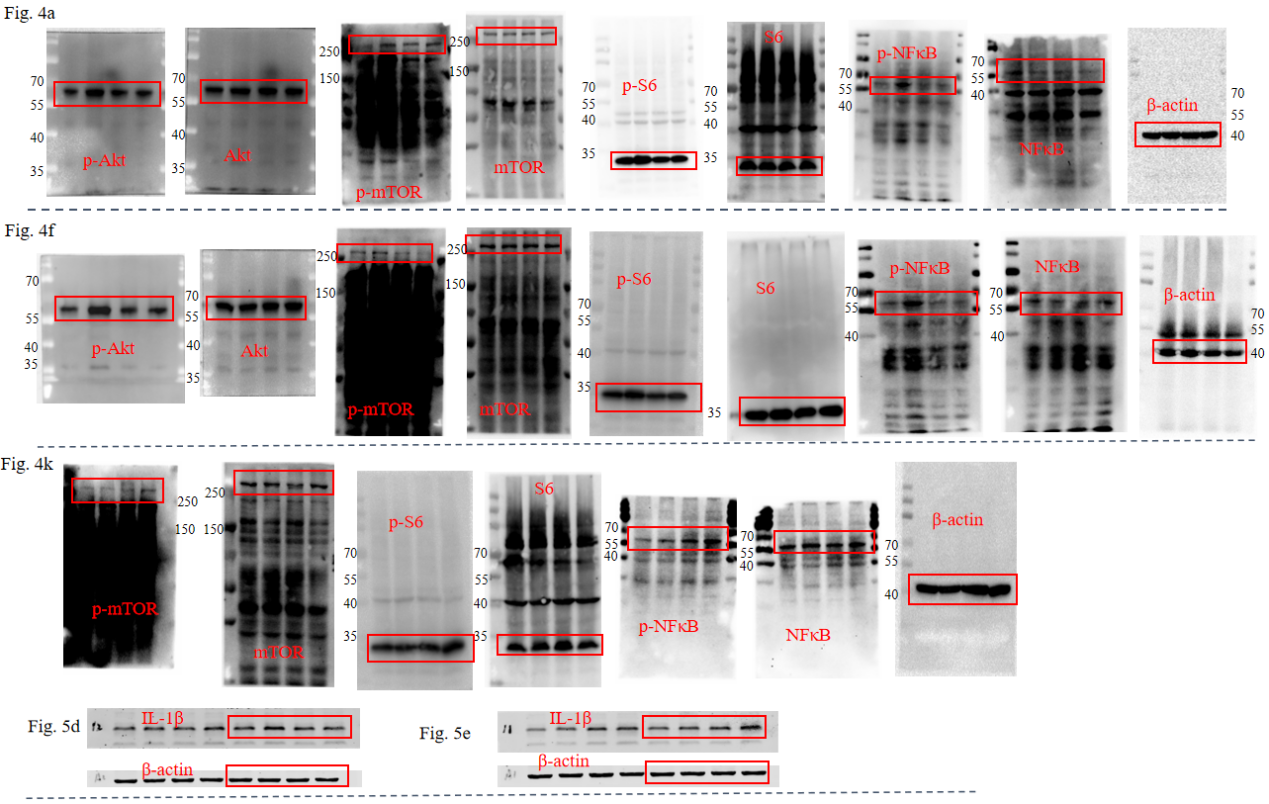


**Supplementary information, Fig. S7 Original blots for Fig. 4a, 4f, 4k, 5d and 5e.**


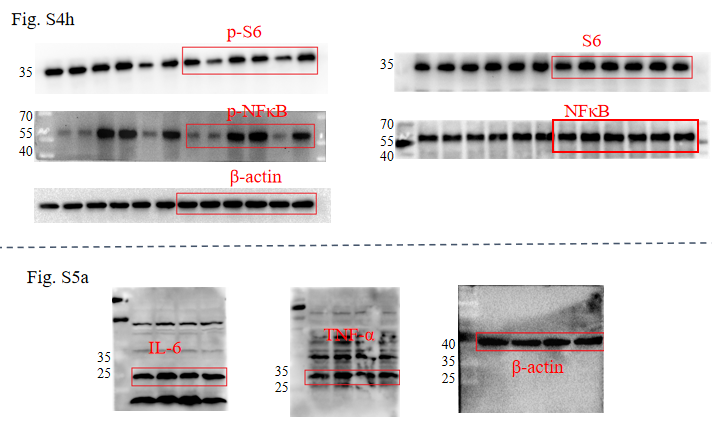


**Supplementary information, Fig. S8 Original blots for Fig. S4h, S5a.**

Supplementary Table 1. The statistical information about this study.

| **Figures and numbers of animals or cells** | **Statistical analysis** | **Post hoc tests** | **mean ± SEM** |  |
| --- | --- | --- | --- | --- |
| 1b: SPT after COS injection  CON+Vehicle (*n* = 7)  CRS+Vehicle (*n* = 7)  CON+COS (*n* = 7)  CRS+COS (*n* = 7) | Two-way ANOVA:  CRS: F (1, 24) = 11.930,  *P* = 0.002  COS: F (1, 24) = 18.703,  *P* = 0.0002  CRS × COS: F (1, 24) = 1.832,  *P* = 0.189 | Sidak's post hoc test  CON + Vehicle vs CRS + Vehicle, *P* < 0.01; CRS + Vehicle vs CRS + COS, *P* < 0.01 | CON+Vehicle 77.09 ± 1.24  CRS+Vehicle 72.211 ± 1.40  CON+COS 80.10 ± 0.55  CRS+COS 77.97 ± 0.57 |  |
| 1c: TST after COS injection  CON+Vehicle (*n* = 7)  CRS+Vehicle (*n* = 7)  CON+COS (*n* = 7)  CRS+COS (*n* = 7) | Two-way ANOVA:  CRS: F (1, 24) = 6.544,  *P* = 0.017  COS: F (1, 24) = 9.211,  *P* = 0.006  CRS × COS: F (1, 24) = 2.764,  *P* = 0.109 | Sidak's post hoc test  CON + Vehicle vs CRS + Vehicle, *P* < 0.05; CRS + Vehicle vs CRS + COS, *P* < 0.01 | CON+Vehicle 105.33 ± 13.19  CRS+Vehicle 155.01 ± 16.76  CON+COS 89.17 ± 7.54  CRS+COS 99.71 ± 6.53 |  |
| 1d: FST after COS injection  CON+Vehicle (*n* = 7)  CRS+Vehicle (*n* = 7)  CON+COS (*n* = 7)  CRS+COS (*n* = 7) | Two-way ANOVA:  CRS: F (1, 24) = 6.076,  *P* = 0.021  COS: F (1, 24) = 11.310,  *P* = 0.003  CRS × COS: F (1, 24) = 2.284,  *P* = 0.144 | Sidak's post hoc test  CON + Vehicle vs CRS + Vehicle, *P* < 0.05; CRS + Vehicle vs CRS + COS, *P* < 0.01 | CON+Vehicle 98.43 ± 9.84  CRS+Vehicle 139.57 ± 10.68  CON+COS 79.29 ± 11.22  CRS+COS 89.14 ± 9.56 |  |
| 1e: ST after COS injection  CON+Vehicle (*n* = 7)  CRS+Vehicle (*n* = 7)  CON+COS (*n* = 7)  CRS+COS (*n* = 7) | Two-way ANOVA:  CRS: F (1, 24) = 6.363,  *P* = 0.002  COS: F (1, 24) = 0.456,  *P* = 0.046  CRS × COS: F (1, 24) = 7.600,  *P* = 0.011 | Sidak's post hoc test  CON + Vehicle vs CRS + Vehicle, *P* < 0.01; CRS + Vehicle vs CRS + COS, *P* < 0.05 | CON+Vehicle 172.00 ± 12.21  CRS+Vehicle 117.29 ± 5.66  CON+COS 150.43 ± 11.51  CRS+COS 152.86 ± 10.77 |  |
| 1f: OFT after COS injection  CON+Vehicle (*n* = 7)  CRS+Vehicle (*n* = 7)  CON+COS (*n* = 7)  CRS+COS (*n* = 7) | Two-way ANOVA:  CRS: F (1, 24) = 0.331,  *P* = 0.570  COS: F (1, 24) = 0.547,  *P* = 0.467  CRS × COS: F (1, 24) = 0.331,  *P* = 0.570 | Sidak's post hoc test  CON + Vehicle vs CRS + Vehicle, *P* > 0.05; CRS + Vehicle vs CRS + COS, *P* > 0.05 | CON+Vehicle 18.43 ± 0.83  CRS+Vehicle 18.43 ± 0.84  CON+COS 19.57 ± 1.02  CRS+COS 18.57 ± 0.75 |  |
| 1h: SPT after COS injection  CON+Vehicle (*n* = 7)  CRS+Vehicle (*n* = 7)  CON+COS (*n* = 7)  CRS+COS (*n* = 7) | Two-way ANOVA:  CRS: F (1, 24) = 6.436,  *P* = 0.018  COS: F (1, 24) = 2.638,  *P* = 0.117  CRS × COS: F (1, 24) = 7.600,  *P* = 0.047 | Sidak's post hoc test  CON + Vehicle vs CRS + Vehicle, *P* < 0.01; CRS + Vehicle vs CRS + COS, *P* < 0.05 | CON+Vehicle 79.27 ± 1.19  CRS+Vehicle 72.40 ± 1.19  CON+COS 78.59 ± 1.94  CRS+COS 77.91 ± 1.50 |  |
| 1i: TST after COS injection  CON+Vehicle (*n* = 7)  CRS+Vehicle (*n* = 7)  CON+COS (*n* = 7)  CRS+COS (*n* = 7) | Two-way ANOVA:  CRS: F (1, 24) = 1.892,  *P* = 0.050  COS: F (1, 24) = 4.898,  *P* = 0.037  CRS × COS: F (1, 24) = 4.257,  *P* = 0.050 | Sidak's post hoc test  CON + Vehicle vs CRS + Vehicle, *P* < 0.05; CRS + Vehicle vs CRS + COS, *P* < 0.05 | CON+Vehicle 100.14 ± 8.33  CRS+Vehicle 155.86 ± 13.99  CON+COS 97.71 ± 20.64  CRS+COS 86.57 ± 18.94 |  |
| 1j: FST after COS injection  CON+Vehicle (*n* = 7)  CRS+Vehicle (*n* = 7)  CON+COS (*n* = 7)  CRS+COS (*n* = 7) | Two-way ANOVA:  CRS: F (1, 24) = 8.530,  *P* = 0.008  COS: F (1, 24) = 0.384,  *P* = 0.013  CRS × COS: F (1, 24) = 11.100,  *P* = 0.003 | Sidak's post hoc test  CON + Vehicle vs CRS + Vehicle, *P* < 0.001; CRS + Vehicle vs CRS + COS, *P* < 0.05 | CON+Vehicle 57.86 ± 10.89  CRS+Vehicle 127.29 ± 12.70  CON+COS 85.00 ± 8.18  CRS+COS 80.43 ± 12.10 |  |
| 1k: ST after COS injection  CON+Vehicle (*n* = 7)  CRS+Vehicle (*n* = 7)  CON+COS (*n* = 7)  CRS+COS (*n* = 7) | Two-way ANOVA:  CRS: F (1, 24) = 4.135,  *P* = 0.053  COS: F (1, 24) = 0.640,  *P* = 0.432  CRS × COS: F (1, 24) = 7.498,  *P* = 0.002 | Sidak's post hoc test  CON + Vehicle vs CRS + Vehicle, *P* < 0.01; CRS + Vehicle vs CRS + COS, *P* < 0.05 | CON+Vehicle 163.57 ± 10.45  CRS+Vehicle 117.14 ± 5.72  CON+COS 144.71 ± 11.87  CRS+COS 151.57 ± 9.79 |  |
| 1l: OFT after COS injection  CON+Vehicle (*n* = 7)  CRS+Vehicle (*n* = 7)  CON+COS (*n* = 7)  CRS+COS (*n* = 7) | Two-way ANOVA:  CRS: F (1, 24) = 0.831,  *P* = 0.371  COS: F (1, 24) = 0.005,  *P* = 0.945  CRS × COS: F (1, 24) = 0.595,  *P* = 0.448 | Sidak's post hoc test  CON + Vehicle vs CRS + Vehicle, *P* > 0.05; CRS + Vehicle vs CRS + COS, *P* > 0.05 | CON+Vehicle 17.00 ± 0.98  CRS+Vehicle 17.14 ± 0.91  CON+COS 16.29 ± 1.27  CRS+COS 18.00 ± 0.87 |  |
| 2b. Iba1^+^CD68^+^ fluorescence intensity of DG  CON+Vehicle (*n* = 3)  CRS+Vehicle (*n* = 3)  CON+COS (*n* = 3)  CRS+COS (*n* = 3) | Two-way ANOVA:  CRS: F (1, 8) = 16.760,  *P* = 0.004  COS: F (1, 8) = 2.757,  *P* = 0.135  CRS × COS: F (1, 8) = 8.47,  *P* = 0.020 | Sidak's post hoc test  CON + Vehicle vs CRS + Vehicle, *P* < 0.01; CRS + Vehicle vs CRS + COS, *P* < 0.05 | CON+Vehicle 1.01 ± 0.01  CRS+Vehicle 1.72 ± 0.15  CON+COS 1.13 ± 0.06  CRS+COS 1.25 ± 0.12 |  |
| 2d. Microglial soma size of DG  CON+Vehicle (*n* = 12)  CRS+Vehicle (*n* = 12)  CON+COS (*n* = 10)  CRS+COS (*n* = 12) | Two-way ANOVA:  CRS: F (1, 42) = 3.57,  *P* = 0.066  COS: F (1, 42) = 6.031,  *P* = 0.018  CRS × COS: F (1, 42) = 4.300,  *P* = 0.044 | Sidak's post hoc test  CON + Vehicle vs CRS + Vehicle, *P* < 0.05; CRS + Vehicle vs CRS + COS, *P* < 0.01 | CON+Vehicle 1.05 ± 0.03  CRS+Vehicle 1.20 ± 0.05  CON+COS 1.04 ± 0.03  CRS+COS 1.03 ± 0.04 |  |
| 2e. Microglial length of DG  CON+Vehicle (*n* = 12)  CRS+Vehicle (*n* = 12)  CON+COS (*n* = 12)  CRS+COS (*n* = 12) | Two-way ANOVA:  CRS: F (1, 44) = 0.889,  *P* = 0.035  COS: F (1, 44) = 2.895,  *P* = 0.096  CRS × COS: F (1, 44) = 6.826,  *P* = 0.012 | Sidak's post hoc test  CON + Vehicle vs CRS + Vehicle, *P* < 0.05; CRS + Vehicle vs CRS + COS, *P* < 0.01 | CON+Vehicle 1016.42 ± 33.45  CRS+Vehicle 893.83 ± 49.21  CON+COS 985.00 ± 31.19  CRS+COS 1042.58 ± 15.53 |  |
| 2f: Number of crossings  CON+Vehicle (*n* = 12)  CRS+Vehicle (*n* = 12)  CON+COS (*n* = 12)  CRS+COS (*n* = 12) | Repeated measures ANOVA  CRS: F (1, 44) = 3.876,  *P* = 0.025  COS: F (1, 44) = 0.179,  *P* = 0.009 |  |  |  |
| 3c:BrdU^+^Sox2^+^GFAP^+^RGLs in the DG of COS-treated mice  CON+Vehicle (*n* = 3)  CRS+Vehicle (*n* = 3)  CON+COS (*n* = 3)  CRS+COS (*n* = 3)  BrdU^+^DCX^+^neuroblasts in DG of COS-treated mice  CON+Vehicle (*n* = 3)  CRS+Vehicle (*n* = 3)  CON+COS (*n* = 3)  CRS+COS (*n* = 3) | Two-way ANOVA:  CRS: F (1, 8) = 46.71,  *P* = 0.0001  COS: F (1, 8) = 11.96,  *P* = 0.009  CRS × COS: F (1, 8) = 10.31,  *P* = 0.012  Two-way ANOVA:  CRS: F (1, 8) = 38.75,  *P* = 0.0003  COS: F (1, 8) = 4.230,  *P* = 0.074  CRS × COS: F (1, 8) = 7.11,  *P* = 0.029 | Sidak's post hoc test  CON + Vehicle vs CRS + Vehicle, *P* < 0.05; CRS + Vehicle vs CRS + COS, *P* < 0.001  Sidak's post hoc test  CON + Vehicle vs CRS + Vehicle, *P* < 0.01; CRS + Vehicle vs CRS + COS, *P* < 0.001 | CON+Vehicle 1066.67 ± 24.04  CRS+Vehicle 660.00 ± 66.58  CON+COS 1076.67 ± 17.64  CRS+COS 930.00 ± 35.12  CON+Vehicle 1136.33 ± 58.77  CRS+Vehicle 703.33 ± 39.30  CON+COS 1106.67 ± 54.87  CRS+COS 933.33 ± 38.44 |  |
| 3e. Dendritic length of DCX^+^ newborn neurons in the DG  CON+Vehicle (*n* = 12)  CRS+Vehicle (*n* = 12)  CON+COS (*n* = 12)  CRS+COS (*n* = 12) | Two-way ANOVA:  CRS: F (1, 44) = 9.377,  *P* = 0.004  COS: F (1, 44) = 31.660,  *P* < 0.0001  CRS × COS: F (1, 44) = 2.344,  *P* = 0.133 | Sidak's post hoc test  CON + Vehicle vs CRS + Vehicle, *P* < 0.001; CRS + Vehicle vs CRS + COS, *P* < 0.01 | CON+Vehicle 648.33 ± 24.80  CRS+Vehicle 548.33 ± 326.11  CON+COS 737.50 ± 16.29  CRS+COS 704.17 ± 318.28 |  |
| 3f: Number of crossings  CON+Vehicle (*n* = 12)  CRS+Vehicle (*n* = 12)  CON+COS (*n* = 12)  CRS+COS (*n* = 12) | Repeated measures ANOVA  CRS: F (1, 44) = 8.247,  *P* = 0.046  COS: F (1, 44) = 1.293,  *P* = 0.035 |  |  |  |
| 3i: BrdU^+^NeuN^+^ neurons in DG of COS-treated mice  CON+Vehicle (*n* = 3)  CRS+Vehicle (*n* = 3)  CON+COS (*n* = 3)  CRS+COS (*n* = 3) | Two-way ANOVA:  CRS: F (1, 8) = 4.898,  *P* = 0.058  COS: F (1, 8) = 3.035,  *P* = 0.120  CRS × COS: F (1, 8) = 4.898,  *P* = 0.058 | Sidak's post hoc test  CON + Vehicle vs CRS + Vehicle, *P* < 0.05; CRS + Vehicle vs CRS + COS, *P* < 0.05 | CON+Vehicle 813.33 ± 20.28  CRS+Vehicle 656.67 ± 63.60  CON+COS 796.67 ± 21.86  CRS+COS 796.67 ± 8.82 |  |
| 4b. p-Akt protein expression in BV2 cells  Vehicle+Vehicle (*n* = 6)  LPS+Vehicle (*n* = 6)  Vehicle+COS (*n* = 6)  LPS+COS (*n* = 6) | Two-way ANOVA:  LPS: F (1, 20) = 1.657,  *P* = 0.213  COS: F (1, 20) = 7.663,  *P* = 0.012  LPS × COS: F (1, 20) = 7.860,  *P* = 0.011 | Sidak's post hoc test  Vehicle + Vehicle vs LPS + Vehicle, *P* < 0.05; LPS + Vehicle vs LPS + COS, *P* < 0.01 | Vehicle+Vehicle 0.99 ± 0.04  LPS+Vehicle 1.19 ± 0.03  Vehicle+COS 0.99 ± 0.02  LPS+COS 0.92 ± 0.08 |  |
| 4c. p-mTOR protein expression in BV2 cells  Vehicle+Vehicle (*n* = 6)  LPS+Vehicle (*n* = 6)  Vehicle+COS (*n* = 6)  LPS+COS (*n* = 6) | Two-way ANOVA:  LPS: F (1, 20) = 9.281,  *P* = 0.006  COS: F (1, 20) = 10.230,  *P* = 0.005  LPS × COS: F (1, 20) = 1.637,  *P* = 0.215 | Sidak's post hoc test  Vehicle + Vehicle vs LPS + Vehicle, *P* < 0.05; LPS + Vehicle vs LPS + COS, *P* < 0.01 | Vehicle+Vehicle 0.99 ± 0.05  LPS+Vehicle 1.23 ± 0.07  Vehicle+COS 0.89 ± 0.06  LPS+COS 0.98 ± 0.03 |  |
| 4d. p-S6 protein expression in BV2 cells  Vehicle+Vehicle (*n* = 6)  LPS+Vehicle (*n* = 6)  Vehicle+COS (*n* = 6)  LPS+COS (*n* = 6) | Two-way ANOVA:  LPS: F (1, 20) = 6.618,  *P* = 0.018  COS: F (1, 20) = 7.165,  *P* = 0.015  LPS × COS: F (1, 20) = 0.947,  *P* = 0.342 | Sidak's post hoc test  Vehicle + Vehicle vs LPS + Vehicle, *P* < 0.05; LPS + Vehicle vs LPS + COS, *P* < 0.05 | Vehicle+Vehicle 0.98 ± 0.03  LPS+Vehicle 1.15 ± 0.04  Vehicle+COS 0.90 ± 0.06  LPS+COS 0.97 ± 0.06 |  |
| 4e. p-NFκB protein expression in BV2 cells  Vehicle+Vehicle (*n* = 6)  LPS+Vehicle (*n* = 6)  Vehicle+COS (*n* = 6)  LPS+COS (*n* = 6) | Two-way ANOVA:  LPS: F (1, 20) = 2.748,  *P* = 0.113  COS: F (1, 20) = 10.210,  *P* = 0.005  LPS × COS: F (1, 20) = 5.208,  *P* = 0.034 | Sidak's post hoc test  Vehicle + Vehicle vs LPS + Vehicle, *P* < 0.05; LPS + Vehicle vs LPS + COS, *P* < 0.01 | Vehicle+Vehicle 0.96 ± 0.05  LPS+Vehicle 1.23 ± 0.06  Vehicle+COS 0.90 ± 0.08  LPS+COS 0.85 ± 0.08 |  |
| 4g. p-Akt protein expression in DG  CON+Vehicle (*n* = 6)  CRS+Vehicle (*n* = 6)  CON+COS (*n* = 6)  CRS+COS (*n* = 6) | Two-way ANOVA:  CRS: F (1, 20) = 4.938,  *P* = 0.038  COS: F (1, 20) = 8.515,  *P* = 0.009  CRS × COS: F (1, 20) = 2.964,  *P* = 0.101 | Sidak's post hoc test  CON + Vehicle vs CRS + Vehicle, *P* < 0.05; CRS + Vehicle vs CRS + COS, *P* < 0.01 | CON+Vehicle 0.98 ± 0.02  CRS+Vehicle 1.12 ± 0.03  CON+COS 0.93 ± 0.05  CRS+COS 0.95 ± 0.04 |  |
| 4h. p-mTOR protein expression in DG  CON+Vehicle (*n* = 6)  CRS+Vehicle (*n* = 6)  CON+COS (*n* = 6)  CRS+COS (*n* = 6) | Two-way ANOVA:  CRS: F (1, 20) = 4.689,  *P* = 0.043  COS: F (1, 20) = 9.636,  *P* = 0.006  CRS × COS: F (1, 20) = 1.580,  *P* = 0.223 | Sidak's post hoc test  CON + Vehicle vs CRS + Vehicle, *P* < 0.05; CRS + Vehicle vs CRS + COS, *P* < 0.05 | CON+Vehicle 1.00 ± 0.05  CRS+Vehicle 1.19 ± 0.05  CON+COS 0.90 ± 0.07  CRS+COS 0.95 ± 0.06 |  |
| 4i. p-S6 protein expression in DG  CON+Vehicle (*n* = 6)  CRS+Vehicle (*n* = 6)  CON+COS (*n* = 6)  CRS+COS (*n* = 6) | Two-way ANOVA:  CRS: F (1, 20) = 4.428,  *P* = 0.048  COS: F (1, 20) = 7.834,  *P* = 0.011  CRS × COS: F (1, 20) = 1.764,  *P* = 0.199 | Sidak's post hoc test  CON + Vehicle vs CRS + Vehicle, *P* < 0.05; CRS + Vehicle vs CRS + COS, *P* < 0.05 | CON+Vehicle 0.99 ± 0.02  CRS+Vehicle 1.13 ± 0.05  CON+COS 0.93 ± 0.06  CRS+COS 0.96 ± 0.02 |  |
| 4j. p-NFκB protein expression in DG  CON+Vehicle (*n* = 6)  CRS+Vehicle (*n* = 6)  CON+COS (*n* = 6)  CRS+COS (*n* = 6) | Two-way ANOVA:  CRS: F (1, 20) = 7.778,  *P* = 0.011  COS: F (1, 20) = 5.859,  *P* = 0.025  CRS × COS: F (1, 20) = 2.249,  *P* = 0.149 | Sidak's post hoc test  CON + Vehicle vs CRS + Vehicle, *P* < 0.05; CRS + Vehicle vs CRS + COS, *P* < 0.05 | CON+Vehicle 0.98 ± 0.06  CRS+Vehicle 1.25 ± 0.04  CON+COS 0.92 ± 0.09  CRS+COS 0.99 ± 0.05 |  |
| 4l. p-mTOR protein expression in DG  CON+COS+Vehicle (*n* = 6)  CRS+COS+Vehicle (*n* = 6)  CON+COS+MHY1485 (*n* = 6)  CRS+COS+MHY1485 (*n* = 6) | Two-way ANOVA:  COS: F (1, 20) = 0.010,  *P* = 0.921  MHY: F (1, 20) = 6.077,  *P* = 0.023  COS × MHY: F (1, 20) = 2.774,  *P* = 0.111 | Sidak's post hoc test  CRS+COS+Vehicle vs CRS+COS+ MHY, *P* < 0.05 | CON+COS+Vehicle 1.02 ± 0.05  CRS+COS+Vehicle 0.94 ± 0.07  CON+COS+MHY 1.06 ± 0.05  CRS+COS+MHY 1.15 ± 0.02 |  |
| 4m. p-S6 protein expression in DG  CON+COS+Vehicle (*n* = 6)  CRS+COS+Vehicle (*n* = 6)  CON+COS+MHY1485 (*n* = 6)  CRS+COS+MHY1485 (*n* = 6) | Two-way ANOVA:  COS: F (1, 20) = 2.810,  *P* = 0.109  MHY: F (1, 20) = 6.216,  *P* = 0.022  COS × MHY: F (1, 20) = 1.057,  *P* = 0.316 | Sidak's post hoc test  CRS+COS+Vehicle vs CRS+COS+ MHY, *P* < 0.05 | CON+COS+Vehicle 1.02 ± 0.03  CRS+COS+Vehicle 1.06 ± 0.02  CON+COS+MHY 1.11 ± 0.03  CRS+COS+MHY 1.27 ± 0.11 |  |
| 4n. p-NFκB protein expression in DG  CON+COS+Vehicle (*n* = 6)  CRS+COS+Vehicle (*n* = 6)  CON+COS+MHY1485 (*n* = 6)  CRS+COS+MHY1485 (*n* = 6) | Two-way ANOVA:  COS: F (1, 20) = 7.930,  *P* = 0.011  MHY: F (1, 20) = 24.320,  *P* < 0.001  COS × NHY: F (1, 20) = 13.140,  *P* = 0.002 | Sidak's post hoc test  CRS+COS+Vehicle vs CRS+COS+ MHY, *P* < 0.001 | CON+COS+Vehicle 1.00 ± 0.02  CRS+COS+Vehicle 0.96 ± 0.04  CON+COS+MHY 1.07 ± 0.05  CRS+COS+MHY 1.42 ± 0.09 |  |
| 4p. Microglial soma size of DG  CON+COS+Vehicle (*n* = 14)  CRS+COS+Vehicle (*n* = 14)  CON+COS+MHY1485 (*n* = 14)  CRS+COS+MHY1485 (*n* = 14) | Two-way ANOVA:  COS: F (1, 52) = 0.455,  *P* = 0.503  MHY: F (1, 52) = 7.222,  *P* = 0.010  COS × MHY: F (1, 52) = 1.791,  *P* = 0.187 | Sidak's post hoc test  CRS + COS + Vehicle vs CRS + COS + MHY,  *P* < 0.05 | CON+COS+Vehicle 1.03 ± 0.07  CRS+COS+Vehicle 0.99 ± 0.04  CON+COS+MHY 1.12 ± 0.06  CRS+COS+MHY 1.24 ± 0.08 |  |
| 4q. Microglial length of DG  CON+COS+Vehicle (*n* = 14)  CRS+COS+Vehicle (*n* = 14)  CON+COS+MHY1485 (*n* = 14)  CRS+COS+MHY1485 (*n* = 14) | Two-way ANOVA:  COS: F (1, 52) = 2.770,  *P* = 0.102  MHY: F (1, 52) = 9.278,  *P* = 0.004  COS × MHY: F (1, 52) = 0.631,  *P* = 0.431 | Sidak's post hoc test  CRS + COS + Vehicle vs CRS + COS + MHY,  *P* < 0.05 | CON+COS+Vehicle 1339.93 ± 60.21  CRS+COS+Vehicle 1282.64 ± 64.81  CON+COS+MHY 1191.71 ± 66.59  CRS+COS+MHY 1029.86 ± 71.24 |  |
| 4r. Number of crossings  CON+COS+Vehicle (*n* = 14)  CRS+COS+Vehicle (*n* = 14)  CON+COS+MHY1485 (*n* = 14)  CRS+COS+MHY1485 (*n* = 14) | Repeated measures ANOVA  COS: F (1, 52) = 3.928,  *P* = 0.134  MHY: F (1, 52) = 0.193,  *P* = 0.009 |  |  |  |
| 5c:BrdU^+^Sox2^+^GFAP^+^RGLs in the DG of MHY1485-treated mice  CON+COS+Vehicle (*n* = 6)  CRS+COS+Vehicle (*n* = 6)  CON+COS+MHY1485 (*n* = 6)  CRS+COS+MHY1485 (*n* = 6)  BrdU^+^DCX^+^neuroblasts in DG of MHY1485-treated mice  CON+COS+Vehicle (*n* = 6)  CRS+COS+Vehicle (*n* = 6)  CON+COS+MHY1485 (*n* = 6)  CRS+COS+MHY1485 (*n* = 6)  BrdU^+^NeuN^+^neurons in DG of MHY1485-treated mice  CON+COS+Vehicle (*n* = 6)  CRS+COS+Vehicle (*n* = 6)  CON+COS+MHY1485 (*n* = 6)  CRS+COS+MHY1485 (*n* = 6) | Two-way ANOVA:  COS: F (1, 20) = 10.510,  *P* = 0.004  MHY: F (1, 20) = 5.864  *P* = 0.025  COS × MHY: F (1, 20) = 4.585,  *P* = 0.045  Two-way ANOVA:  COS: F (1, 20) = 13.690,  *P* = 0.001  MHY: F (1, 20) = 18.560  *P* = 0.0003  COS × MHY: F (1, 20) = 4.556,  *P* = 0.045  Two-way ANOVA:  COS: F (1, 20) = 26.420,  *P* < 0.0001  MHY: F (1, 20) = 19.040  *P* = 0.0003  COS × MHY: F (1, 20) = 6.371,  *P* = 0.020 | Sidak's post hoc test  CRS + COS + Vehicle vs CRS + COS + MHY,  *P* < 0.05  Sidak's post hoc test  CRS + COS + Vehicle vs CRS + COS + MHY,  *P* < 0.01  Sidak's post hoc test  CRS + COS + Vehicle vs CRS + COS + MHY,  *P* < 0.001 | CON+COS+Vehicle 1261.17 ± 30.01  CRS+COS+Vehicle 1182.67 ± 78.44  CON+COS+MHY 1241.17 ± 66.37  CRS+COS+MHY 857.33 ± 94.21  CON+COS+Vehicle 1455.50 ± 21.74  CRS+COS+Vehicle 1354.17 ± 82.32  CON+COS+MHY 1314.83 ± 28.05  CRS+COS+MHY 937.17 ± 93.40  CON+COS+Vehicle 834.83 ± 35.70  CRS+COS+Vehicle 742.17 ± 25.26  CON+COS+MHY 769.67 ± 22.74  CRS+COS+MHY 498.17 ± 50.90 |  |
| 5d. IL-1β protein expression in DG of COS-treated mice  CON+Vehicle (*n* = 6)  CRS+Vehicle (*n* = 6)  CON+COS (*n* = 6)  CRS+COS (*n* = 6) | Two-way ANOVA:  CON: F (1, 20) = 3.638,  *P* = 0.071  COS: F (1, 20) = 5.346  *P* = 0.032  CRS × COS: F (1, 20) = 4.574,  *P* = 0.045 | Sidak's post hoc test  CON + Vehicle vs CRS + Vehicle,  *P* < 0.05  CRS + Vehicle vs CRS + COS,  *P* < 0.05 | CON+Vehicle 1.03 ± 0.02  CRS+Vehicle 1.14 ± 0.05  CON+COS 1.02 ± 0.02  CRS+COS 1.01 ± 0.02 |  |
| 5e. IL-1β protein expression in DG of MHY1485-treated mice  CON+COS+Vehicle (*n* = 6)  CRS+COS+Vehicle (*n* = 6)  CON+COS+MHY1485 (*n* = 6)  CRS+COS+MHY1485 (*n* = 6) | Two-way ANOVA:  COS: F (1, 20) = 12.420,  *P* = 0.002  MHY: F (1, 20) = 3.016  *P* = 0.098  COS × MHY: F (1, 20) = 1.503,  *P* = 0.235 | Sidak's post hoc test  CRS + COS + Vehicle vs CRS + COS + MHY,  *P* < 0.01 | CON+COS+Vehicle 1.01 ± 0.01  CRS+COS+Vehicle 1.08 ± 0.03  CON+COS+MHY 1.02 ± 0.03  CRS+COS+MHY 1.18 ± 0.05 |  |
| 5g:BrdU^+^Sox2^+^GFAP^+^RGLs in the DG of IL-1β-treated mice  CON+COS+Vehicle (*n* = 6)  CRS+COS+Vehicle (*n* = 6)  CON+COS+IL-1β (*n* = 6)  CRS+COS+IL-1β (*n* = 6)  BrdU^+^DCX^+^neuroblasts in DG of IL-1β-treated mice  CON+COS+Vehicle (*n* = 6)  CRS+COS+Vehicle (*n* = 6)  CON+COS+IL-1β (*n* = 6)  CRS+COS+IL-1β (*n* = 6)  BrdU^+^NeuN^+^neurons in DG of IL-1β5-treated mice  CON+COS+Vehicle (*n* = 6)  CRS+COS+Vehicle (*n* = 6)  CON+COS+IL-1β (*n* = 6)  CRS+COS+IL-1β (*n* = 6) | Two-way ANOVA:  COS: F (1, 20) = 30.930,  *P* < 0.0001  IL-1β: F (1, 20) = 9.097,  *P* = 0.007  COS × IL-1β: F (1, 20) = 7.667,  *P* = 0.012  Two-way ANOVA:  COS: F (1, 20) = 9.234,  *P* = 0.007  IL-1β: F (1, 20) = 19.110,  *P* = 0.0003  COS × IL-1β: F (1, 20) = 4.237,  *P* = 0.053  Two-way ANOVA:  COS: F (1, 20) = 5.513,  *P* = 0.029  IL-1β: F (1, 20) = 9.193,  *P* = 0.007  COS × IL-1β: F (1, 20) = 2.126,  *P* = 0.160 | Sidak's post hoc test  CRS + COS + Vehicle vs CRS + COS + IL-1β,  *P* < 0.01  Sidak's post hoc test  CRS + COS + Vehicle vs CRS + COS +IL-1β,  *P* < 0.01  Sidak's post hoc test  CRS + COS + Vehicle vs CRS + COS + IL-1β,  *P* < 0.05 | CON+COS+Vehicle 1224.83 ± 83.18  CRS+COS+Vehicle 1015.83 ± 35.01  CON+COS+IL-1β 1206.33 ± 73.07  CRS+COS+IL-1β  582.83 ± 94.47  CON+COS+Vehicle 1170.67 ± 67.08  CRS+COS+Vehicle 1107.17 ± 55.41  CON+COS+IL-1β 1020.83 ± 84.60  CRS+COS+IL-1β 690.67 ± 45.36  CON+COS+Vehicle 1140.67 ± 65.56  CRS+COS+Vehicle 1078.00 ± 69.06  CON+COS+IL-1β 1029.83 ± 42.55  CRS+COS+IL-1β 761.83 ± 94.64 |  |
| 6b: SPT of MHY1485-treated mice  CON+COS+Vehicle (*n* = 7)  CRS+COS+Vehicle (*n* = 7)  CON+COS+MHY1485 (*n* = 7)  CRS+COS+MHY1485 (*n* = 7) | Two-way ANOVA:  COS: F (1, 24) = 1.506,  *P* = 0.232  MHY: F (1, 24) = 6.236,  *P* = 0.020  COS × MHY: F (1, 24) = 2.604,  *P* = 0.120 | Sidak's post hoc test  CRS + COS + Vehicle vs CRS + COS + MHY,  *P* < 0.05 | CON+COS+Vehicle 73.91 ± 1.19  CRS+COS+Vehicle 75.01 ± 1.19  CON+COS+MHY 71.40 ± 2.13  CRS+COS+MHY 63.31 ± 5.01 |  |
| 6c: TST of MHY1485-treated mice  CON+COS+Vehicle (*n* = 7)  CRS+COS+Vehicle (*n* = 7)  CON+COS+MHY1485 (*n* = 7)  CRS+COS+MHY1485 (*n* = 7) | Two-way ANOVA:  COS: F (1, 24) = 7.461,  *P* = 0.012  MHY: F (1, 24) = 5.298,  *P* = 0.030  COS × MHY: F (1, 24) = 1,384,  *P* = 0.251 | Sidak's post hoc test  CRS + COS + Vehicle vs CRS + COS + MHY,  *P* < 0.05 | CON+COS+Vehicle 103.66 ± 11.49  CRS+COS+Vehicle 121.34 ± 8.61  CON+COS+MHY 116.46 ± 13.19  CRS+COS+MHY 160.90 ± 11.72 |  |
| 6d: FST of MHY1485-treated mice  CON+COS+Vehicle (*n* = 7)  CRS+COS+Vehicle (*n* = 7)  CON+COS+MHY1485 (*n* = 7)  CRS+COS+MHY1485 (*n* = 7) | Two-way ANOVA:  COS: F (1, 24) = 3.727,  *P* = 0.065  MHY: F (1, 24) = 2.391,  *P* = 0.135  COS × MHY: F (1, 24) = 3.367,  *P* = 0.079 | Sidak's post hoc test  CRS + COS + Vehicle vs CRS + COS + MHY,  *P* < 0.05 | CON+COS+Vehicle 92.54 ± 14.65  CRS+COS+Vehicle 94.10 ± 14.63  CON+COS+MHY 87.84 ± 22.37  CRS+COS+MHY 149.16 ± 11.46 |  |
| 6e: ST of MHY1485-treated mice  CON+COS+Vehicle (*n* = 7)  CRS+COS+Vehicle (*n* = 7)  CON+COS+MHY1485 (*n* = 7)  CRS+COS+MHY1485 (*n* = 7) | Two-way ANOVA:  COS: F (1, 24) = 3.473,  *P* = 0.075  MHY: F (1, 24) = 6.604,  *P* = 0.017  COS × MHY: F (1, 24) = 1.041,  *P* = 0.318 | Sidak's post hoc test  CRS + COS + Vehicle vs CRS + COS + MHY,  *P* < 0.05 | CON+COS+Vehicle 163.47 ± 11.36  CRS+COS+Vehicle 154.60 ± 11.11  CON+COS+MHY 147.17 ± 12.13  CRS+COS+MHY 116.83 ± 6.57 |  |
| 6g: SPT of IL-1β-treated mice  CON+COS+Vehicle (*n* = 7)  CRS+COS+Vehicle (*n* = 7)  CON+COS+IL-1β (*n* = 7)  CRS+COS+IL-1β (*n* = 7) | Two-way ANOVA:  COS: F (1, 24) = 2.180,  *P* = 0.153  IL-1β: F (1, 24) = 2.296,  *P* = 0.143  COS × IL-1β: F (1, 24) = 3.924,  *P* = 0.059 | Sidak's post hoc test  CRS + COS + Vehicle vs CRS + COS + IL-1β,  *P* < 0.05 | CON+COS+Vehicle 73.94 ± 1.65  CRS+COS+Vehicle 75.63 ± 1.12  CON+COS+IL-1β 75.50 ± 1.97  CRS+COS+IL-1β 63.94 ± 6.07 |  |
| 6h: TST of IL-1β-treated mice  CON+COS+Vehicle (*n* = 7)  CRS+COS+Vehicle (*n* = 7)  CON+COS+IL-1β (*n* = 7)  CRS+COS+IL-1β (*n* = 7) | Two-way ANOVA:  COS: F (1, 24) = 0.031,  *P* = 0.862  IL-1β: F (1, 24) = 20.780,  *P* = 0.0001  COS × IL-1β: F (1, 24) = 2.235,  *P* = 0.148 | Sidak's post hoc test  CRS + COS + Vehicle vs CRS + COS + IL-1β,  *P* < 0.001 | CON+COS+Vehicle 86.90 ± 8.19  CRS+COS+Vehicle 74.41 ± 7.30  CON+COS+IL-1β 115.90 ± 13.90  CRS+COS+IL-1β 131.71 ± 6.69 |  |
| 6i: FST of IL-1β-treated mice  CON+COS+Vehicle (*n* = 7)  CRS+COS+Vehicle (*n* = 7)  CON+COS+IL-1β (*n* = 7)  CRS+COS+IL-1β (*n* = 7) | Two-way ANOVA:  COS: F (1, 24) = 1.698,  *P* = 0.205  IL-1β: F (1, 24) = 21.170,  *P* = 0.0001  COS × IL-1β: F (1, 24) = 4.056,  *P* = 0.055 | Sidak's post hoc test  CRS + COS + Vehicle vs CRS + COS + IL-1β,  *P* < 0.001 | CON+COS+Vehicle 58.97 ± 13.24  CRS+COS+Vehicle 51.80 ± 9.67  CON+COS+IL-1β 85.07 ± 8.64  CRS+COS+IL-1β 118.53 ± 7.97 |  |
| 6j: ST of IL-1β-treated mice  CON+COS+Vehicle (*n* = 7)  CRS+COS+Vehicle (*n* = 7)  CON+COS+IL-1β (*n* = 7)  CRS+COS+IL-1β (*n* = 7) | Two-way ANOVA:  COS: F (1, 24) = 2.234,  *P* = 0.148  IL-1β: F (1, 24) = 8.963,  *P* = 0.006  COS × IL-1β: F (1, 24) = 0.277,  *P* = 0.604 | Sidak's post hoc test  CRS + COS + Vehicle vs CRS + COS + IL-1β,  *P* < 0.05 | CON+COS+Vehicle 144.04 ± 8.83  CRS+COS+Vehicle 135.74 ± 10.14  CON+COS+IL-1β 122.90 ± 7.88  CRS+COS+IL-1β 105.59 ± 7.12 |  |
| S1b: FST after COS (2.5 μM) treatment  CON+Vehicle (*n* = 7)  CRS+Vehicle (*n* = 7)  CON+COS (*n* = 7)  CRS+COS (*n* = 7) | Two-way ANOVA:  CRS: F (1, 24) = 17.900,  *P* = 0.0003  COS: F (1, 24) = 2.091,  *P* = 0.161  CRS × COS: F (1, 24) = 1.618,  *P* = 0.216 | Sidak's post hoc test  CRS + Vehicle vs CRS + Vehicle,  *P* < 0.01  CRS + Vehicle vs CRS + COS,  *P* > 0.05 | CON+Vehicle 93.43 ± 10.11  CRS+Vehicle 152.14 ± 10.23  CON+COS 91.57 ± 8.78  CRS+COS 123.14 ± 13.09 |  |
| S1c: FST after COS (5 μM) treatment  CON+Vehicle (*n* = 7)  CRS+Vehicle (*n* = 7)  CON+COS (*n* = 7)  CRS+COS (*n* = 7) | Two-way ANOVA:  CRS: F (1, 24) = 18.320,  *P* = 0.0003  COS: F (1, 24) = 2.193,  *P* = 0.152  CRS × COS: F (1, 24) = 4.394,  *P* = 0.047 | Sidak's post hoc test  CRS + Vehicle vs CRS + Vehicle,  *P* < 0.001  CRS + Vehicle vs CRS + COS,  *P* < 0.05 | CON+Vehicle 93.43 ± 10.11  CRS+Vehicle 152.14 ± 10.23  CON+COS 91.57 ± 8.78  CRS+COS 123.14 ± 13.09 |  |
| S1e: SPT after COS treatment  Vehicle+Vehicle (*n* = 6)  LPS+Vehicle (*n* = 6)  Vehicle+COS (*n* = 6)  LPS+COS (*n* = 6) | Two-way ANOVA:  LPS: F (1, 20) = 4.677,  *P* = 0.043  COS: F (1, 20) = 4.512,  *P* = 0.046  LPS × COS: F (1, 20) = 5.723,  *P* = 0.027 | Sidak's post hoc test  CON + Vehicle vs LPS + Vehicle,  *P* < 0.01  LPS + Vehicle vs LPS + COS,  *P* < 0.01 | Vehicle+Vehicle 77.38 ± 0.96  LPS+Vehicle 67.47 ± 3.58  Vehicle+COS 76.80 ± 1.47  LPS+COS 77.30 ± 1.75 |  |
| S1f: TST after COS treatment  Vehicle+Vehicle (*n* = 6)  LPS+Vehicle (*n* = 6)  Vehicle+COS (*n* = 6)  LPS+COS (*n* = 6) | Two-way ANOVA:  LPS: F (1, 20) = 1.341,  *P* = 0.261  COS: F (1, 20) = 8.432,  *P* = 0.009  LPS × COS: F (1, 20) = 10.560,  *P* = 0.004 | Sidak's post hoc test  CON + Vehicle vs LPS + Vehicle,  *P* < 0.05  LPS + Vehicle vs LPS + COS,  *P* < 0.001 | Vehicle+Vehicle 104.17 ± 6.66  LPS+Vehicle 146.67 ± 8.14  Vehicle+COS 107.50 ± 12.72  LPS+COS 87.33 ± 9.98 |  |
| S1g: FST after COS treatment  Vehicle+Vehicle (*n* = 6)  LPS+Vehicle (*n* = 6)  Vehicle+COS (*n* = 6)  LPS+COS (*n* = 6) | Two-way ANOVA:  LPS: F (1, 20) = 1.911,  *P* = 0.182  COS: F (1, 20) = 5.717,  *P* = 0.027  LPS × COS: F (1, 20) = 10.090,  *P* = 0.005 | Sidak's post hoc test  CON + Vehicle vs LPS + Vehicle,  *P* < 0.01  LPS + Vehicle vs LPS + COS,  *P* < 0.01 | Vehicle+Vehicle 71.67 ± 11.88  LPS+Vehicle 129.67 ± 8.98  Vehicle+COS 81.67 ± 16.18  LPS+COS 58.83 ± 12.80 |  |
| S1h: ST after COS treatment  Vehicle+Vehicle (*n* = 6)  LPS+Vehicle (*n* = 6)  Vehicle+COS (*n* = 6)  LPS+COS (*n* = 6) | Two-way ANOVA:  LPS: F (1, 20) = 5.724,  *P* = 0.027  COS: F (1, 20) = 3.595,  *P* = 0.073  LPS × COS: F (1, 20) = 2.537,  *P* = 0.127 | Sidak's post hoc test  CON + Vehicle vs LPS + Vehicle,  *P* < 0.05  LPS + Vehicle vs LPS + COS,  *P* < 0.05 | Vehicle+Vehicle 149.83 ± 14.38  LPS+Vehicle 101.67 ± 9.35  Vehicle+COS 153.50 ± 14.91  LPS+COS 143.83 ± 8.23 |  |
| S1j: FST after COS (5 mg/kg) treatment  CON+Vehicle (*n* = 5)  CRS+Vehicle (*n* = 5)  CON+COS (*n* = 5)  CRS+COS (*n* = 5) | Two-way ANOVA:  CRS: F (1, 16) = 1.677,  *P* = 0.214  COS: F (1, 16) = 0.267,  *P* = 0.613  CRS × COS: F (1, 16) = 9.013,  *P* = 0.108 | Sidak's post hoc test  CRS + Vehicle vs CRS + Vehicle,  *P* < 0.05  CRS + Vehicle vs CRS + COS,  *P* > 0.05 | CON+Vehicle 60.00 ± 14.64  CRS+Vehicle 117.40 ± 7.94  CON+COS 70.40 ± 14.53  CRS+COS 93.20 ± 15.01 |  |
| S1k: FST after COS (10 mg/kg) treatment  CON+Vehicle (*n* = 5)  CRS+Vehicle (*n* = 5)  CON+COS (*n* = 5)  CRS+COS (*n* = 5) | Two-way ANOVA:  CRS: F (1, 16) = 0.715,  *P* = 0.410  COS: F (1, 16) = 2.947,  *P* = 0.105  CRS × COS: F (1, 16) = 19.060,  *P* = 0.105 | Sidak's post hoc test  CRS + Vehicle vs CRS + Vehicle,  *P* < 0.05  CRS + Vehicle vs CRS + COS,  *P* > 0.05 | CON+Vehicle 88.60 ± 6.44  CRS+Vehicle 129.00 ± 14.63  CON+COS 59.20 ± 14.15  CRS+COS 119.00 ± 8.43 |  |
| S1l: FST after COS (20 mg/kg) treatment  CON+Vehicle (*n* = 5)  CRS+Vehicle (*n* = 5)  CON+COS (*n* = 5)  CRS+COS (*n* = 5) | Two-way ANOVA:  CRS: F (1, 16) = 2.245,  *P* = 0.154  COS: F (1, 16) = 4.424,  *P* = 0.049  CRS × COS: F (1, 16) = 9.596,  *P* = 0.007 | Sidak's post hoc test  CRS + Vehicle vs CRS + Vehicle,  *P* < 0.05  CRS + Vehicle vs CRS + COS,  *P* < 0.05 | CON+Vehicle 72.60 ± 14.22  CRS+Vehicle 141.00 ± 12.67  CON+COS 63.60 ± 19.34  CRS+COS 87.40 ± 12.22 |  |
| S2b: Relative Iba1^+^ fluorescence intensity after COS incubation  Vehicle+Vehicle (*n* = 15)  LPS+Vehicle (*n* = 15)  Vehicle+COS (*n* = 15)  LPS+COS (*n* = 15) | Two-way ANOVA:  LPS: F (1, 56) = 12.430,  *P* = 0.0008  COS: F (1, 56) = 4.021,  *P* = 0.050  LPS × COS: F (1, 56) = 5.839,  *P* = 0.019 | Sidak's post hoc test  Vehicle + Vehicle vs LPS+ Vehicle, *P* < 0.05; LPS + Vehicle vs LPS + COS, *P* < 0.05 | Vehicle+Vehicle 1.00 ± 0.12  LPS+Vehicle 2.70 ± 0.53  Vehicle+COS 1.12 ± 0.09  LPS+COS 1.43 ± 0.16 |  |
| S2b: Relative CD68^+^ fluorescence intensity after COS incubation  Vehicle+Vehicle (*n* = 15)  LPS+Vehicle (*n* = 15)  Vehicle+COS (*n* = 15)  LPS+COS (*n* = 15) | Two-way ANOVA:  LPS: F (1, 56) = 26.490,  *P* < 0.0001  COS: F (1, 56) = 5.490,  *P* = 0.023  LPS × COS: F (1, 56) = 1.557,  *P* = 0.217 | Sidak's post hoc test  Vehicle + Vehicle vs LPS+ Vehicle, *P* < 0.05; LPS + Vehicle vs LPS + COS, *P* < 0.001 | Vehicle+Vehicle 1.00 ± 0.13  LPS+Vehicle 2.15 ± 0.27  Vehicle+COS 0.80 ± 0.12  LPS+COS 1.50 ± 0.16 |  |
| S3b:BrdU^+^Sox2^+^GFAP^+^RGLs in the DG of CRS  CON (*n* = 3)  CRS (*n* = 3)  BrdU^+^DCX^+^neuroblasts in DG of CRS  CON (*n* = 3)  CRS (*n* = 3) | Unpaired *t* test:  *t* = 5.556, *P* < 0.01  Unpaired *t* test:  *t* = 5.359, *P* < 0.01 |  | CON 961.70 ± 25.35  CRS 561.90 ± 14.39  CON 833.70 ± 10.42  CRS 570.70 ± 9.48 |  |
| S4b: Relative Iba1^+^ fluorescence intensity after COS incubation  Vehicle+Vehicle (*n* = 6)  LPS+Vehicle (*n* = 6)  Vehicle+COS (*n* = 6)  LPS+COS (*n* = 6) | Two-way ANOVA:  LPS: F (1, 56) = 20.770,  *P* < 0.0001  COS: F (1, 56) = 23.530,  *P* < 0.0001  LPS × COS: F (1, 56) = 6.115,  *P* = 0.017 | Sidak's post hoc test  Vehicle + Vehicle vs LPS + Vehicle, *P* < 0.001; LPS + Vehicle vs LPS + COS, *P* < 0.001 | Vehicle+Vehicle 1.00 ± 0.19  LPS+Vehicle 2.53 ± 0.30  Vehicle+COS 0.48 ± 0.19  LPS+COS 0.94 ± 0.18 |  |
| S4b: Relative pS6^+^ fluorescence intensity after COS incubation  Vehicle+Vehicle (*n* = 6)  LPS+Vehicle (*n* = 6)  Vehicle+COS (*n* = 6)  LPS+COS (*n* = 6) | Two-way ANOVA:  LPS: F (1, 56) = 15.360,  *P* = 0.0002  COS: F (1, 56) = 31.650,  *P* < 0.0001  LPS × COS: F (1, 56) = 14.060,  *P* = 0.0004 | Sidak's post hoc test  Vehicle + Vehicle vs LPS + Vehicle, *P* < 0.001; LPS + Vehicle vs LPS + COS, *P* < 0.001 | Vehicle+Vehicle 1.00 ± 0.07  LPS+Vehicle 1.79 ± 0.17  Vehicle+COS 0.81 ± 0.05  LPS+COS 0.83 ± 0.09 |  |
| S4d: Relative Iba1^+^ fluorescence intensity after COS incubation  Vehicle+Vehicle (*n* = 6)  LPS+Vehicle (*n* = 6)  Vehicle+COS (*n* = 6)  LPS+COS (*n* = 6) | Two-way ANOVA:  LPS: F (1, 56) = 31.610,  *P* < 0.0001  COS: F (1, 56) = 11.890,  *P* = 0.001  LPS × COS: F (1, 56) = 10.240,  *P* < 0.0001 | Sidak's post hoc test  Vehicle + Vehicle vs LPS+ Vehicle, *P* < 0.001; LPS + Vehicle vs LPS + COS, *P* < 0.001 | Vehicle+Vehicle 1.00 ± 0.22  LPS+Vehicle 3.29 ± 0.35  Vehicle+COS 0.94 ± 0.13  LPS+COS 1.56 ± 0.28 |  |
| S4d: Relative pNFκB^+^ fluorescence intensity after COS incubation  Vehicle+Vehicle (*n* = 6)  LPS+Vehicle (*n* = 6)  Vehicle+COS (*n* = 6)  LPS+COS (*n* = 6) | Two-way ANOVA:  LPS: F (1, 20) = 48.210,  *P* < 0.0001  COS: F (1, 20) = 45.280,  *P* < 0.0001  LPS × COS: F (1, 20) = 43.580,  *P* < 0.0001 | Sidak's post hoc test  Vehicle + Vehicle vs LPS + Vehicle, *P* < 0.001; LPS + Vehicle vs LPS + COS, *P* < 0.001 | Vehicle+Vehicle 1.00 ± 0.13  LPS+Vehicle 4.05 ± 0.40  Vehicle+COS 0.97 ± 0.12  LPS+COS 1.05 ± 0.14 |  |
| S4f: Relative pNFκB^+^ fluorescence intensity in nucleus after COS incubation  Vehicle+Vehicle (*n* = 15)  LPS+Vehicle (*n* = 15)  Vehicle+COS (*n* = 15)  LPS+COS (*n* = 15) | Two-way ANOVA:  LPS: F (1, 56) = 11.87,  *P* < 0.001  COS: F (1, 56) = 8.44,  *P* < 0.005  LPS × COS: F (1, 56) = 22.07,  *P* < 0.0001 | Sidak's post hoc test  Vehicle + Vehicle vs LPS + Vehicle, *P* < 0.0001; LPS + Vehicle vs LPS + COS, *P* < 0.0001 | Vehicle+Vehicle 1.00 ± 0.29  LPS+Vehicle 3.09 ± 0.32  Vehicle+COS 1.21 ± 0.19  LPS+COS 1.52 ± 0.25 |  |
| S4g: Cellular viability  Vehicle+Vehicle+Vehicle (*n* = 6)  Vehicle+Vehicle+MHY (*n* = 6)  Vehicle+COS+MHY (*n* = 6)  LPS+Vehicle+Vehicle (*n* = 6)  LPS+COS+Vehicle (*n* = 6)  LPS+COS+MHY (*n* = 6) | Two-way ANOVA:  LPS: F (1, 20) = 9.07,  *P* > 0.05  COS: F (1, 20) = 7.38,  *P* > 0.05  LPS × COS: F (1, 20) = 10.45,  *P* > 0.05  LPS × MHY: F (1, 20) = 10.48,  *P* > 0.05 | Sidak's post hoc test  Vehicle+Vehicle+Vehicle vs LPS+Vehicle+ Vehicle, *P* > 0.05; LPS+Vehicle+ Vehicle vs LPS+COS+Vehicle, *P* > 0.05; LPS+COS+Vehicle vs LPS+COS+MHY, *P* > 0.05 | Vehicle+Vehicle+Vehicle 99.07 ± 0.64  Vehicle+Vehicle+MHY 98.42 ± 0.82  Vehicle+COS+MHY 98.76 ± 0.05  LPS+Vehicle+ Vehicle 99.09 ± 0.45  LPS+COS+Vehicle 98.67 ± 0.06  LPS+COS+MHY 98.09 ± 0.19 |  |
| S4i: p-S6/S6 protein expression in BV2 cells of MHY1485-treated mice  Vehicle+Vehicle+Vehicle (*n* = 6)  Vehicle+Vehicle+MHY (*n* = 6)  Vehicle+COS+MHY (*n* = 6)  LPS+Vehicle+Vehicle (*n* = 6)  LPS+COS+Vehicle (*n* = 6)  LPS+COS+MHY (*n* = 6) | Two-way ANOVA:  LPS: F (1, 20) = 0.394,  *P* < 0.05  COS: F (1, 20) = 0.195,  *P* < 0.01  LPS × COS: F (1, 20) = 1.09,  *P* < 0.01  LPS × MHY: F (1, 20) = 0.92,  *P* < 0.01 | Sidak's post hoc test  Vehicle+Vehicle+Vehicle vs LPS+Vehicle+ Vehicle, *P* < 0.05; LPS+Vehicle+ Vehicle vs LPS+COS+Vehicle, *P* < 0.01; LPS+COS+Vehicle vs LPS+COS+MHY, *P* < 0.01 | Vehicle+Vehicle+Vehicle 1.00 ± 0.03  Vehicle+Vehicle+MHY 0.95 ± 0.84  Vehicle+COS+MHY 1.209 ± 0.09  LPS+Vehicle+ Vehicle 1.398 ± 0.74  LPS+COS+Vehicle 0.77 ± 0.06  LPS+COS+MHY 1.14 ± 0.75 |  |
| S4i: S6/β-actin protein expression in BV2 cells of MHY1485-treated mice  Vehicle+Vehicle+Vehicle (*n* = 6)  Vehicle+Vehicle+MHY (*n* = 6)  Vehicle+COS+MHY (*n* = 6)  LPS+Vehicle+Vehicle (*n* = 6)  LPS+COS+Vehicle (*n* = 6)  LPS+COS+MHY (*n* = 6) | Two-way ANOVA:  LPS: F (1, 20) = 9.042,  *P* > 0.05  COS: F (1, 20) = 4.593,  *P* > 0.05  LPS × COS: F (1, 20) = 9.422,  *P* > 0.05  LPS × MHY: F (1, 20) = 0.485,  *P* > 0.05 | Sidak's post hoc test  Vehicle+Vehicle+Vehicle vs LPS+Vehicle+ Vehicle, *P* > 0.05; LPS+Vehicle+ Vehicle vs LPS+COS+Vehicle, *P* > 0.05; LPS+COS+Vehicle vs LPS+COS+MHY, *P* > 0.05 | Vehicle+Vehicle+Vehicle 0.99 ± 0.13  Vehicle+Vehicle+MHY 1.04 ± 0.49  Vehicle+COS+MHY 0.97 ± 0.12  LPS+Vehicle+ Vehicle 1.398 ± 0.74  LPS+COS+Vehicle 0.94 ± 0.38  LPS+COS+MHY 0.89 ± 0.09 |  |
| S4j: p-NF-κB/NF-κB protein expression in BV2 cells of MHY1485-treated mice  Vehicle+Vehicle+Vehicle (*n* = 6)  Vehicle+Vehicle+MHY (*n* = 6)  Vehicle+COS+MHY (*n* = 6)  LPS+Vehicle+Vehicle (*n* = 6)  LPS+COS+Vehicle (*n* = 6)  LPS+COS+MHY (*n* = 6) | Two-way ANOVA:  LPS: F (1, 20) = 0.123,  *P* < 0.001  COS: F (1, 20) = 0.531,  *P* < 0.001  LPS × COS: F (1, 20) = 0.942,  *P* < 0.001  LPS × MHY: F (1, 20) = 0.125,  *P* < 0.001 | Sidak's post hoc test  Vehicle+Vehicle+Vehicle vs LPS+Vehicle+ Vehicle, *P* < 0.001; LPS+Vehicle+ Vehicle vs LPS+COS+Vehicle, *P* < 0.001; LPS+COS+Vehicle vs LPS+COS+MHY, *P* < 0.001 | Vehicle+Vehicle+Vehicle 1.00 ± 0.01  Vehicle+Vehicle+MHY 0.94 ± 0.53  Vehicle+COS+MHY 1.47 ± 0.24  LPS+Vehicle+ Vehicle 1.43 ± 0.19  LPS+COS+Vehicle 0.84 ± 0.15  LPS+COS+MHY 1.23 ± 0.10 |  |
| S4j: NF-κB/β-actin protein expression in BV2 cells of MHY1485-treated mice  Vehicle+Vehicle+Vehicle (*n* = 6)  Vehicle+Vehicle+MHY (*n* = 6)  Vehicle+COS+MHY (*n* = 6)  LPS+Vehicle+Vehicle (*n* = 6)  LPS+COS+Vehicle (*n* = 6)  LPS+COS+MHY (*n* = 6) | Two-way ANOVA:  LPS: F (1, 20) = 9.042,  *P* > 0.05  COS: F (1, 20) = 9.084,  *P* > 0.05  LPS × COS: F (1, 20) = 9.443,  *P* > 0.05  LPS × MHY: F (1, 20) = 4.938,  *P* > 0.05 | Sidak's post hoc test  Vehicle+Vehicle+Vehicle vs LPS+Vehicle+ Vehicle, *P* > 0.05; LPS+Vehicle+ Vehicle vs LPS+COS+Vehicle, *P* > 0.05; LPS+COS+Vehicle vs LPS+COS+MHY, *P* > 0.05 | Vehicle+Vehicle+Vehicle 0.95 ± 0.17  Vehicle+Vehicle+MHY 0.94 ± 0.03  Vehicle+COS+MHY 0.94 ± 0.84  LPS+Vehicle+ Vehicle 0.92 ± 0.09  LPS+COS+Vehicle 0.91 ± 0.44  LPS+COS+MHY 0.95 ± 0.13 |  |
| S5b: IL-6 protein expression in DG of CRS-exposed mice  CON+Vehicle (*n* = 6)  CRS+Vehicle (*n* = 6)  CON+COS (*n* = 6)  CRS+COS (*n* = 6) | Two-way ANOVA:  CRS: F (1, 20) = 0.555,  *P* = 0.046  COS: F (1, 20) = 4.511,  *P* = 0.285  CRS × COS: F (1, 20) = 11.650,  *P* = 0.465 | Sidak's post hoc test  CRS + Vehicle vs CRS + Vehicle,  *P* < 0.05  CRS + Vehicle vs CRS + COS,  *P* > 0.05 | CON+Vehicle 0.98 ± 0.09  CRS+Vehicle 1.27 ± 0.07  CON+COS 0.86 ± 0.10  CRS+COS 1.06 ± 0.07 |  |
| S5c: TNF-α protein expression in DG of CRS-exposed mice  CON+Vehicle (*n* = 6)  CRS+Vehicle (*n* = 6)  CON+COS (*n* = 6)  CRS+COS (*n* = 6) | Two-way ANOVA:  CRS: F (1, 20) = 27.56,  *P* < 0.0001  COS: F (1, 20) = 6.891,  *P* = 0.0562  CRS × COS: F (1, 20) = 0.191,  *P* = 0.666 | Sidak's post hoc test  CRS + Vehicle vs CRS + Vehicle,  *P* < 0.01  CRS + Vehicle vs CRS + COS,  *P* > 0.05 | CON+Vehicle 1.02 ± 0.05  CRS+Vehicle 1.28 ± 0.06  CON+COS 0.86 ± 0.04  CRS+COS 1.16 ± 0.06 |  |
| S6b: SPT of MHY1485-treated mice  CON+COS+Vehicle (*n* = 6)  CRS+COS+Vehicle (*n* = 6)  CON+COS+MHY1485 (*n* = 6)  CRS+COS+MHY1485 (*n* = 6) | Two-way ANOVA:  COS: F (1, 20) = 12.38,  *P* = 0.002  MHY: F (1, 20) = 3.121,  *P* = 0.093  COS × MHY: F (1, 20) = 1.563,  *P* = 0.226 | Sidak's post hoc test  CRS + COS + Vehicle vs CRS + COS + MHY,  *P* < 0.01 | CON+COS+Vehicle 81.52 ± 3.48  CRS+COS+Vehicle 75.00 ± 1.67  CON+COS+MHY 80.03 ± 2.80  CRS+COS+MHY 66.33 ± 3.21 |  |
| S6c: TST of MHY1485-treated mice  CON+COS+Vehicle (*n* = 6)  CRS+COS+Vehicle (*n* = 6)  CON+COS+MHY1485 (*n* = 6)  CRS+COS+MHY1485 (*n* = 6) | Two-way ANOVA:  COS: F (1, 20) = 2.461,  *P* = 0.132  MHY: F (1, 20) = 7.130,  *P* = 0.015  COS × MHY: F (1, 20) = 6.679,  *P* = 0.018 | Sidak's post hoc test  CRS + COS + Vehicle vs CRS + COS + MHY,  *P* < 0.01 | CON+COS+Vehicle 123.90 ± 8.31  CRS+COS+Vehicle 111.27 ± 21.42  CON+COS+MHY 124.97 ± 6.13  CRS+COS+MHY 176.63 ± 7.31 |  |
| S6d: FST of MHY1485-treated mice  CON+COS+Vehicle (*n* = 6)  CRS+COS+Vehicle (*n* = 6)  CON+COS+MHY1485 (*n* = 6)  CRS+COS+MHY1485 (*n* = 6) | Two-way ANOVA:  COS: F (1, 20) = 3.482,  *P* = 0.077  MHY: F (1, 20) = 21.970,  *P* = 0.0001  COS × MHY: F (1, 20) = 0.048,  *P* = 0.828 | Sidak's post hoc test  CRS + COS + Vehicle vs CRS + COS + MHY,  *P* < 0.05 | CON+COS+Vehicle 55.20 ± 16.22  CRS+COS+Vehicle 72.58 ± 9.46  CON+COS+MHY 102.38 ± 4.79  CRS+COS+MHY 124.42 ± 8.42 |  |
| S6e: ST of MHY1485-treated mice  CON+COS+Vehicle (*n* = 6)  CRS+COS+Vehicle (*n* = 6)  CON+COS+MHY1485 (*n* = 6)  CRS+COS+MHY1485 (*n* = 6) | Two-way ANOVA:  COS: F (1, 20) = 8.279,  *P* = 0.009  MHY: F (1, 20) = 3.839,  *P* = 0.064  COS × MHY: F (1, 20) = 6.549,  *P* = 0.019 | Sidak's post hoc test  CRS + COS + Vehicle vs CRS + COS + MHY,  *P* < 0.01 | CON+COS+Vehicle 141.22 ± 9.97  CRS+COS+Vehicle 138.32 ± 8.91  CON+COS+MHY 146.68 ± 9.96  CRS+COS+MHY 97.13 ± 7.39 |  |
| S6g: SPT of IL-1β-treated mice  CON+COS+Vehicle (*n* = 6)  CRS+COS+Vehicle (*n* = 6)  CON+COS+IL-1β (*n* = 6)  CRS+COS+IL-1β (*n* = 6) | Two-way ANOVA:  COS: F (1, 20) = 1.049,  *P* = 0.318  IL-1β: F (1, 20) = 9.671,  *P* = 0.006  COS × IL-1β: F (1, 20) = 0.217,  *P* = 0.647 | Sidak's post hoc test  CRS + COS + Vehicle vs CRS + COS + IL-1β,  *P* < 0.05 | CON+COS+Vehicle 77.07 ± 1.86  CRS+COS+Vehicle 76.07 ± 2.18  CON+COS+IL-1β 72.33 ± 1.58  CRS+COS+IL-1β 69.67 ± 1.46 |  |
| S6h: TST of IL-1β-treated mice  CON+COS+Vehicle (*n* = 6)  CRS+COS+Vehicle (*n* = 6)  CON+COS+IL-1β (*n* = 6)  CRS+COS+IL-1β (*n* = 6) | Two-way ANOVA:  COS: F (1, 20) = 0.305,  *P* = 0.587  IL-1β: F (1, 20) = 2.99,  *P* = 0.099  COS × IL-1β: F (1, 20) = 13.81,  *P* = 0.001 | Sidak's post hoc test  CRS + COS + Vehicle vs CRS + COS + IL-1β,  *P* < 0.01 | CON+COS+Vehicle 114.13 ± 4.95  CRS+COS+Vehicle 81.92 ± 13.21  CON+COS+IL-1β 93.92 ± 6.64  CRS+COS+IL-1β 137.38 ± 13.11 |  |
| S6i: FST of IL-1β-treated mice  CON+COS+Vehicle (*n* = 6)  CRS+COS+Vehicle (*n* = 6)  CON+COS+IL-1β (*n* = 6)  CRS+COS+IL-1β (*n* = 6) | Two-way ANOVA:  COS: F (1, 20) = 3.482,  *P* = 0.077  IL-1β: F (1, 20) = 21.970,  *P* = 0.0001  COS × IL-1β: F (1, 20) = 0.048,  *P* = 0.828 | Sidak's post hoc test  CRS + COS + Vehicle vs CRS + COS + IL-1β,  *P* < 0.01 | CON+COS+Vehicle 55.20 ± 16.22  CRS+COS+Vehicle 72.58 ± 9.46  CON+COS+IL-1β 102.38 ± 4.79  CRS+COS+IL-1β 124.42 ± 8.42 |  |
| S6j: ST of IL-1β-treated mice  CON+COS+Vehicle (*n* = 6)  CRS+COS+Vehicle (*n* = 6)  CON+COS+IL-1β (*n* = 6)  CRS+COS+IL-1β (*n* = 6) | Two-way ANOVA:  COS: F (1, 20) = 4.235,  *P* = 0.053  IL-1β: F (1, 20) = 8.151,  *P* = 0.010  COS × IL-1β: F (1, 20) = 3.564,  *P* = 0.074 | Sidak's post hoc test  CRS + COS + Vehicle vs CRS + COS + IL-1β,  *P* < 0.01 | CON+COS+Vehicle 121.33 ± 9.79  CRS+COS+Vehicle 119.68 ± 7.48  CON+COS+IL-1β 111.95 ± 12.73  CRS+COS+IL-1β 73.67 ± 7.91 |  |
